# CD38 Deficiency Ameliorates Chronic Graft-*Versus*-Host Disease Murine Lupus *via* a B-Cell-Dependent Mechanism

**DOI:** 10.3389/fimmu.2021.713697

**Published:** 2021-08-24

**Authors:** África Martínez-Blanco, Marilú Domínguez-Pantoja, María Botía-Sánchez, Sonia Pérez-Cabrera, Nerea Bello-Iglesias, Paula Carrillo-Rodríguez, Natividad Martin-Morales, Antonio Lario-Simón, María M. Pérez-Sánchez-Cañete, Laura Montosa-Hidalgo, Salvador Guerrero-Fernández, Victoria M. Longobardo-Polanco, Sandra Redondo-Sánchez, Alberto Cornet-Gomez, María Torres-Sáez, Ana Fernández-Ibáñez, Laura Terrón-Camero, Eduardo Andrés-León, Francisco O’Valle, Ramón Merino, Mercedes Zubiaur, Jaime Sancho

**Affiliations:** ^1^Department of Cellular Biology and Immunology, Institute of Parasitology and Biomedicine López-Neyra (IPBLN), Consejo Superior de Investigaciones Científicas (CSIC), Granada, Spain; ^2^Department of Pathology, Faculty of Medicine, University of Granada (UGR), Granada, Spain; ^3^Proteomics Unit, IPBLN, CSIC, Granada, Spain; ^4^Flow Cytometry Unit, IPBLN, CSIC, Granada, Spain; ^5^Microscopy Unit, IPBLN, CSIC, Granada, Spain; ^6^Scientific Instrumentation Center (CIC), UGR, Granada, Spain; ^7^Bioinformatics Unit, IPBLN, CSIC, Granada, Spain; ^8^Department of Molecular and Cellular Signalling, Instituto de Biomedicina y de Biotecnología de Cantabria (IBBTEC), Consejo Superior de Investigaciones Científicas-Universidad de Cantabria (CSIC-UC), Santander, Spain

**Keywords:** CD38, cGVHD lupus-like, T-bet^+^ B cells, anti-ssDNA antibodies, GC B cells, STAT1, type I IFN-signature, inflammation

## Abstract

The absence of the mouse cell surface receptor CD38 in *Cd38^−/−^* mice suggests that this receptor acts as a positive regulator of inflammatory and autoimmune responses. Here, we report that, in the context of the chronic graft-*versus*-host disease (cGVHD) lupus inducible model, the transfer of B6.C-H2bm12/KhEg(bm12) spleen cells into co-isogenic *Cd38^−/−^* B6 mice causes milder lupus-like autoimmunity with lower levels of anti-ssDNA autoantibodies than the transfer of bm12 spleen cells into WT B6 mice. In addition, significantly lower percentages of Tfh cells, as well as GC B cells, plasma cells, and T-bet^+^CD11c^hi^ B cells, were observed in *Cd38^−/−^* mice than in WT mice, while the expansion of Treg cells and Tfr cells was normal, suggesting that the ability of *Cd38^−/−^* B cells to respond to allogeneic help from bm12 CD4^+^ T cells is greatly diminished. The frequencies of T-bet^+^CD11c^hi^ B cells, which are considered the precursors of the autoantibody-secreting cells, correlate with anti-ssDNA autoantibody serum levels, IL-27, and sCD40L. Proteomics profiling of the spleens from WT cGVHD mice reflects a STAT1-driven type I IFN signature, which is absent in *Cd38^−/−^* cGVHD mice. Kidney, spleen, and liver inflammation was mild and resolved faster in *Cd38^−/−^* cGVHD mice than in WT cGVHD mice. We conclude that CD38 in B cells functions as a modulator receptor that controls autoimmune responses.

## Introduction

Systemic lupus erythematosus or SLE is an incurable systemic autoimmune disease, which occurs predominantly in women of childbearing age, characterized by the production of autoantibodies that are deposited as immunocomplexes in various organs (1). The SLE courses with periods of activity and remission, resulting in accumulated damage over time (2). It is a very heterogeneous disease from a clinical point of view and difficult to follow (3). Hence, there is a need of experimental lupus models to investigate the molecular mechanisms of the disease and to discover new biomarkers that better reflect the pathogenesis and activity of the disease and which may be of diagnostic or therapeutic interest (4).

CD38 has an enormous potential to be used as a target to inhibit the chronic inflammatory reactions linked to aging and autoimmunity and to eliminate autoreactive plasma cells that produce potentially harmful autoantibodies (5–7). Increased CD38 expression greatly affects cellular metabolism by lowering intracellular NAD^+^ levels and decreasing NAD-dependent deacetylation performed by sirtuins (8). Thus, high levels of CD38 lead to decreased CD8 T-cell-mediated cytotoxicity and increased propensity to infections in patients with SLE (9). The relevance to lupus is that abnormal NAD-dependent deacetylation could be reverted pharmacologically or with anti-CD38 therapy (10–12). In lupus models, this could also be approached by analyzing the functional effect of CD38 deficiency (13–16). Using the pristane lupus model, we have demonstrated the crucial role of CD38 in promoting aberrant inflammation and lupus-like autoimmunity *via* an apoptosis-driven mechanism, which requires TRPM2 expression (13).

A chronic graft-*versus*-host reaction (cGVHD) induced in non-autoimmune C57BL6 mice (B6) by the adoptive transfer of Ia-incompatible bm12 spleen cells results in a syndrome that closely resembles SLE in the spectrum of autoantibodies and immunopathology (17). In the cGVHD lupus model, the key cellular mechanism that results in the loss of B-cell tolerance is the interaction of donor bm12 CD4^+^ T cells with MHC class II on host B6 B-cell surface, inducing high levels of circulating anti-nuclear antibodies, concomitantly with large frequencies of T follicular helper (Tfh) cells, germinal center (GC) B cells, and plasma cells (18, 19). During T- and B-cell interactions, allogeneic donor CD4^+^ T cells provide the abnormal T cell help to the host B cells, which act as efficient APCs, and the host B cells further augment the clonal expansion, differentiation, and survival of pathogenic T cells. Therefore, this is a suitable model to study the role of CD38 in autoreactive B cells using CD38-deficient mice *versus* B6 WT mice as recipients.

## Material and Methods

### Mice

C57BL/6J (B6) (RRID: IMSR_JAX:000664) WT female mice were from Charles River. B6(C)-H2-Ab1bm12/KhEgJ (bm12) (RRID: IMSR_JAX:001162) female mice were from the Jackson Laboratory. B6.129P2-Cd38tm1Lnd/J (*Cd38^−/−^*) (RRID: IMSR_JAX:003727) female mice were backcrossed for 12 generations to the C57BL/6J (B6) background and were bred and maintained under specific pathogen-free conditions at the IPBLN-CSIC Animal Facility in Granada, Spain. The experimental procedures in animals at IPBLN-CSIC, Spain, were approved by the Institutional Animal Care and Use Committee. The procedures follow the ARRIVE guidelines (20) in accordance with the U.K. Animals (Scientific Procedures Act, 1986) and associated guidelines (EU Directive 2010/63/EU for animal experiments) and with the National Institutes of Health Guide for the Care and Use of Laboratory Animals (NIH Publications No. 8023, revised 1978).

### The bm12-Inducible Model of Systemic Lupus Erythematosus in C57BL/6 Mice

We adapted the bm12 transfer model, as originally described by Morris et al. (21) and modified by Klarquist and Janssen (18). Eight- to 18-week-old B6 WT or *Cd38^−/−^* female mice were injected i.p. with 50–70 × 10^6^ spleen cells from bm12 female mice. Alternatively, bm12 female mice were injected i.p. with 50–70 × 10^6^ spleen cells from WT or *Cd38^−/−^* female mice.

### Flow Cytometry

To block non-specific Fc binding, single-cell suspensions of spleens were incubated with anti-mouse CD16/32 (BD Biosciences Cat# 553142, RRID: AB_394657), 1:200 dilution for 30–60 min, on ice in staining media (1× PBS, w/o calcium or magnesium, 0.5% BSA, 2 mM EDTA). Cells were then separated in different panels and stained with the following anti-mouse antibodies: CD4-PercP (1:250 dilution) (BD Biosciences Cat# 561090, RRID: AB_10562560), TCR-β-FITC (1:500 dilution) (BD Biosciences Cat# 553170, RRID: AB_394682), CXCR5-biotin (1:350 dilution) (BD Biosciences Cat# 551960, RRID: AB_394301), PD1-APC (1:350) (BD Biosciences Cat# 562671, RRID: AB_2737712), CD19-PE-CF594 (1:350 dilution) (BD Biosciences Cat# 562329, RRID: AB_11154580), CD138-PE (1:350 dilution) (BD Biosciences Cat# 561070, RRID: AB_2033998), B220-PE (1:350 dilution) (BD Biosciences Cat# 553089, RRID: AB_394619), CD95-Alexa Fluor 647 (1:350 dilution) (BD Biosciences Cat# 563647, RRID: AB_2738346), GL7-FITC (1:350 dilution) (BD Biosciences Cat# 562080, RRID: AB_10894953), CD38-FITC (1:350 dilution) (BD Biosciences Cat# 558813, RRID: AB_397126), CD19-APC (1:350 dilution) (BD Biosciences Cat# 550992, RRID: AB_398483), CD183-VioBright FITC (1:50 dilution) (Miltenyi Biotec Cat# 130-111-092, RRID: AB_2655744), CD11c-PE (1:50 dilution) (Miltenyi Biotec Cat# 130-110-701, RRID: AB_2654708), and Streptavidin-PE (1:350) (BioLegend Cat# 405203). Flow cytometry analyses at IPBLN-CSIC were performed as previously described (13, 14). Ten thousand to 200,000 events per sample were acquired either in a FACS Calibur flow cytometer (BD Biosciences, RRID: SCR_000401) or FACS Symphony (BD Biosciences) and analyzed with FlowJo software v10.7.1 (BD Biosciences) (FlowJo, RRID: SCR_008520). Absolute cell numbers were calculated by multiplying the percentages of each cell type, referred to as live and singlet cells, with the total number of live spleen cells/mouse counted immediately after isolation by the Trypan blue method. Gating strategies for the different cell subsets are shown in **Figures S1**–**S4**.

### Intracellular T-bet or FoxP3 Staining

After cell surface staining, cells were washed, fixed, and permeabilized using the FoxP3 Staining Buffer Set (Miltenyi Biotec Cat# 130-093-142) and stained with anti-mouse antibodies either T-bet-APC (1:50 dilution) (Miltenyi Biotec Cat# 130-119-783, RRID: AB_2784464) or FoxP3-Alexa Fluor 488 (BD Biosciences Cat# 560407, RRID: AB_1645193) (1:250 dilution). Cells were then washed twice in Perm/Wash buffer (Miltenyi Biotec Cat# 130-093-142), resuspended in staining media, and analyzed by flow cytometry.

### Cytokine Multiplex

The Bio-Plex Pro Mouse Cytokine Th17 kit assay custom 7-Plex (BioRad Cat#LJ00000163, Standard Lot#: 64298607) and 10-Plex kit assay (BioRad Cat#12010828, Standard Lot#64298607) were used to simultaneously test cytokines IL-17F, IL-21, IL-22, IL-23, IL-31, IL-33, and MIP-3α (7-Plex) and IL-17F, IL-21, IL-22, IL-23, IL-25, IL-27, IL-31, IL-33, sCD40L, and MIP-3α (10-Plex). The assay Bio-Plex Pro™ Mouse Cytokine Th17 Panel A 6-Plex Cat#M6000007NY was used to simultaneously quantify cytokines IFN-γ, IL-1β, IL-6, IL-10, IL-17A, and TNF-α. Assays were performed according to the protocols of the manufacturers. Analyses of experimental data were carried out using five-parameter logistic curve fitting in a Luminex 200 (Luminex 100 or 200 Flow Cytometry Analyzer System, RRID: SCR_018025) and in a Bio-Plex (BioRad, RRID: SCR_018026).

### Histopathological Study

For conventional morphology, buffered 10% formaldehyde-fixed, paraffin-embedded longitudinal mice kidney, liver, and spleen sections in sagittal plane were deparaffinized in xylol and rehydrated in ethanol of decreasing gradation. Tissue sections were stained with hematoxylin–eosin (H&E), Masson’s trichrome (MT), and periodic acid-Schiff (PAS). The presence of glomerular lesions (glomerulosclerosis, mesangium increase, crescent, immunocomplexes, and cells per glomerulus) was assessed in at least 100 glomeruli. Tubulo-interstitial damage (brush border loss, tubular dilation, tubular atrophy, hyaline casts, tubular necrosis, tubular mitoses, fibrosis, and inflammatory infiltrate) was also studied. Injury was graded according to Shih et al. (22). A semiquantitative scale of 0 to 3.0 was considered: 0, normal; 0.5, small focal areas of damage; 1, involvement of less than 10% of the cortex; 2, involvement of 10% to 25% of the cortex; and 3, involvement above 25% of the cortex. A morphological study was done in a blinded fashion (FO'V and NMM) on 3-μm sections with light microscopy, using the most appropriate stain for each lesion. The presence of steatosis, hydropic degeneration, inflammatory infiltrate, cholestasis, mitosis, apoptosis, and cell binucleation was assessed in liver sections using a semiquantitative scale of 0 to 4. A millimeter scale in the eyepiece of a microscope (BH2 Olympus (LabX, RRID: SCR_020338) with 40% objective was used to count the leucocyte subset per mm^2^ in the spleen.

### Serological Studies

Serum levels of IgG anti-ssDNA autoantibodies were measured by ELISA, and the results were expressed in titration units (U/ml) as previously described (23, 24). Total IgG serum levels were measured by ELISA as previously described (23, 24).

### Cellular Lysates

Isolated spleen cell count and viability was done on a hemocytometer with 1:1 Trypan blue solution (Sigma-Aldrich Cat# T8154). The spleen cells were washed thoroughly three times with cold 1× PBS, pH 7.6, and 2 mM EDTA and filter sterilized in 0.22 μm filter. The spleen cells were lysed 30 min on ice, as previously described (24–26): 100 μl 1× lysis buffer/10 million cells; the 1× lysis buffer composition is as follows: 150 mM NaCl, 20 mM HEPES (pH: 7.6), 50 mM sodium fluoride, 1 mM EGTA, 0.5% NP-40, and small peptide inhibitors (SPI) (stock, 50×) (25); 1 mM sodium orthovanadate, 10 mM iodoacetamide, and 1 mM PMSF, as phosphatases inhibitors; plus 0.25 µM trichostatin and 5 mM nicotinamide as acetylase inhibitors. Micro BCA Protein Assay (ThermoFisher Cat# 23235) was used for the analysis of the protein concentration of 17,000×*g*-clarified spleen cell lysates.

### Mass Spectrometry Proteomics and Data Processing

Thirty to 40 μg of protein from spleen cell lysates in 1× Laemmli sample buffer was loaded on 4%–20% prestained gels (BioRad Cat# 4568095). Electrophoresis was done at 200 V, in 25 mM TrizMa OH, 192 mM glycine, and pH 8.3, 0.1% SDS buffer, until the whole sample volume was introduced into the gel matrix. The proteins in the gel were visualized by the exposure of the gel during 1 to 5 min to UV light in a GelDoc EZ Image (BioRad). In addition, gel was stained with SYPRO Ruby (BioRad Cat# 1703126) according to the protocol of the manufacturers. An EXQuest Spot Cutter (BioRad) with the PDQuest Advanced program was used to cut the protein bands of the gel.

Protein extracts were analyzed by liquid chromatography‐tandem mass spectrometry (LC‐MS/MS) (Amazon Speed, Bruker) at IPBLN-CSIC Proteomic Facility as described previously (26). Protein identification was done with ProteinScape 4.0 (Bruker) and MASCOT 2.4 data searching using the SwissProt database. For label-free proteomic quantification, we used the exponentially modified protein abundance index (emPAI) implemented into the MASCOT data searching platform without any additional experimental steps. In order to compare between different samples, it is required to normalize emPAI values from MASCOT search to the sum of all emPAI values. Thus, the protein content in molar fraction percentage (M%) can be calculated using the following formula: Protein content (M%)  =  emPAI/Σ(emPAI), where Σ(emPAI) is the summation of emPAI values for all the identified proteins (27). Two biological samples per mouse type and three technical replicates per biological sample were analyzed [The mass spectrometry proteomics data have been deposited to the ProteomeXchange Consortium *via* the PRIDE (28) partner repository with the dataset identifier PXD026947 and 10.6019/PXD026947]. We used ClueGO_v2.5.8 (29) and CluePedia_v1.5.8 (30) within the Cytoscape_v3.8.2 software environment (31) for functional enrichment analysis of the lists of identified proteins. Results are visualized as networks in which Gene Ontology (GO) terms and pathways are grouped based on their biological role. CluePedia allows to expand ClueGO terms into nested networks with associated genes.

### Bioinformatics Analysis

To reduce the dimensionality of the data and to represent the samples based on the amount of variance they contain, a principal component analysis (PCA) was performed to integrate the data from all experiments. A total of 58 mice were used for this purpose: those with complete experimental data for the 11 monitored variables (see **Table S1** in the **Supplementary Material** the total number of experiments and mice used per experiment): for the first group of samples, “NT,” *n* = 8 mice—4 WT and 4 KOs; for the second week, *n* = 18 mice—11 WT and 7 KOs; for the fourth week, *n* = 17—7 WT and 10 KOs. Finally, for the eighth week, *n* = 15 mice, of which 7 were WT and 8 were KO. First, for each time point, we used the turkey_mc_up function from the bigutils package to infer possible outliers (32). It should be noted that it is difficult to distinguish between a mouse with a value that classifies it as an outlier and a mouse with a high response. Therefore, to classify a mouse as an outlier in the total set of variables, it should be an outlier in several variables. As an example, we found that WT13-4 appears as a marginal outlier in the CD4^+^ T, Tfh, and PC variables. Therefore, we decided to keep it. Besides, the vast majority of mice were not marked as outliers for any variable. Then, values were scaled, and subsequently, plots were generated using the factoextra, ggfortify, and cluster R packages (33–35).

### Statistics

Statistical analyses were performed using the GraphPad Prism 9 software (GraphPad Prism, RRID: SCR_002798), using statistical tests as indicated in the text. Statistical significance was visualized as follows: ns = not significant (*P* > 0.05), * = *P* < 0.05, ** = *P* < 0.01, *** = *P* < 0.001, **** = *P* < 0.0001. All experiments have been done using three or more mice and representative images have been chosen for the figures.

## Results

### Long-Term Decreased Frequencies and Numbers of Tfh Cells in *Cd38^−/−^* Mice After Adoptive Transfer of bm12 Spleen Cells

**Figure 1:**
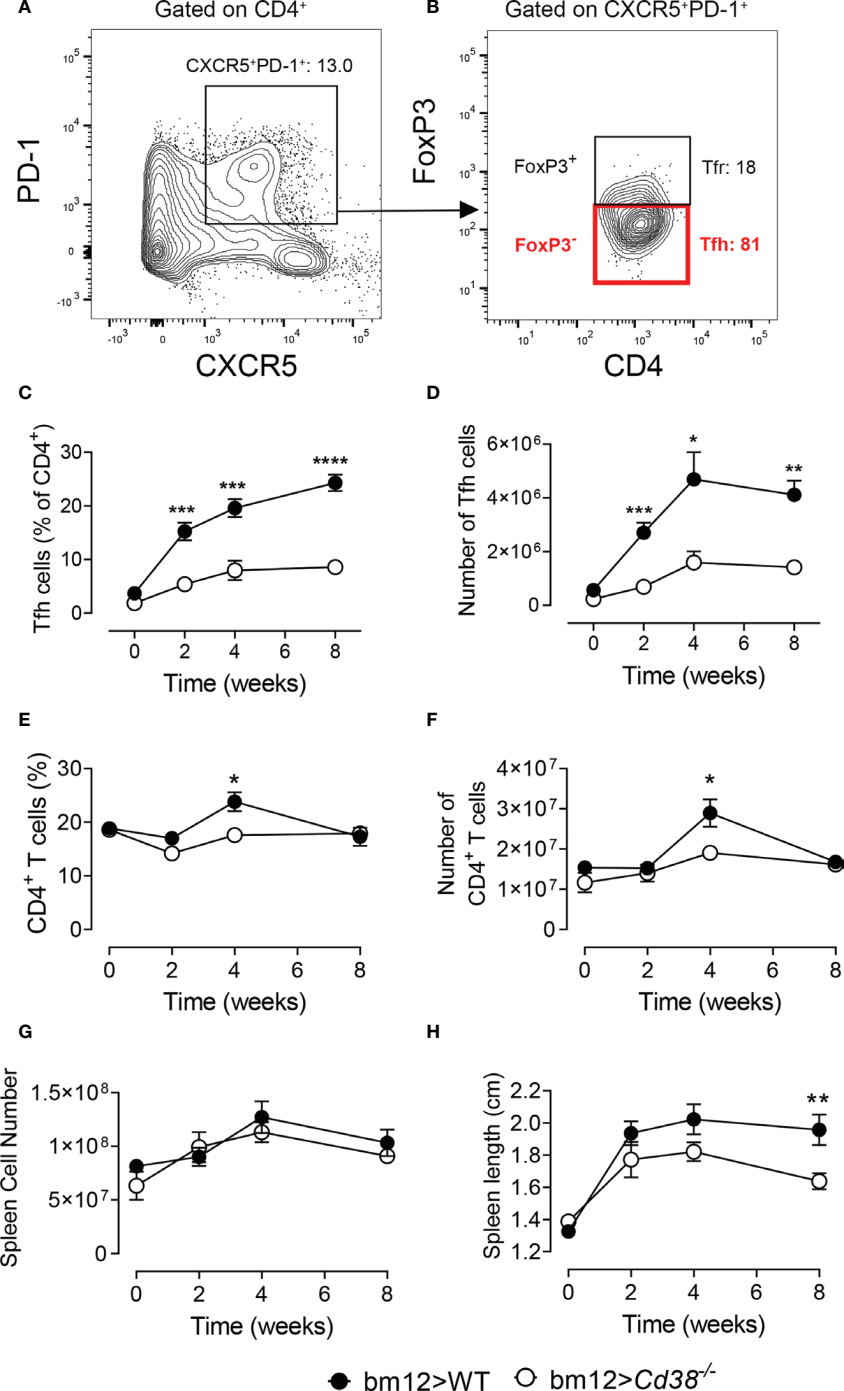
Long-term decreased frequencies and numbers of T follicular helper (Tfh) cells in *Cd38^−/−^* mice after adoptive transfer of bm12 spleen cells. WT mice (closed circles) or *Cd38^−/−^* mice (open circles) were i.p. injected with bm12 cells and euthanized at the indicated time points and spleens were analyzed. **(A, B)** Representative flow cytometry contour plots showing gating strategy for Tfh cells. **(A)** First, gating on CD4^+^ cells, then gating on CXCR5^+^PD-1^+^. **(B)** Then, gating on FoxP3^−^ cells: CD4^+^PD-1^hi^CXCR5^+^FoxP3^−^. **(C)** Percentages of Tfh cells (of CD4^+^ T cells) in spleen at the indicated time points. **(D)** Total number of Tfh cells (in spleen) over time. **(E)** Percentages of CD4^+^ T cells (CD4^+^TCR-β^+^) in spleens. **(F)** Total number of CD4^+^ T cells in spleen. **(G)** Total number of spleen cells over time. **(H)** Spleen length (cm) over time upon the adoptive transfer. In all panels, the symbols are the mean values and the vertical bars represent ± SEM. *P*-values are shown for Welch’s *t*-test. The results are cumulative data from two to four different experiments per time point and mouse type, each with three to five mice per experiment. *P < 0.05, **P < 0.01, ***P < 0.001, ****P < 0.0001.

Selection, isotype switching, and expansion of GC B cells require critical signals from Tfh cells. The adoptive transfer of bm12 lymphocytes into WT B6 mice (WT cGVHD) leads to the expansion of donor-derived Tfh, expansion of recipient-derived GC B cells and plasma cells, and production of ANAs including anti-dsDNA, anti-ssDNA, anti-chromatin, and anti-RBC antibodies (17). We assessed whether the lack of CD38 expression on B cells impairs the expansion of Tfh cells. To this end, 2, 4, and 8 weeks after transferring bm12 CD38-suficient spleen cells into *Cd38^−/−^* mice (*Cd38^−/−^* cGVHD), the frequencies and total numbers of Tfh cells (FoxP3^−^PD-1^hi^CXCR5^+^ within CD4^+^ cells) were assessed in spleen and compared with basal levels (**Figures 1A, B** **)**. The whole gating strategy is shown in **Figure S1**. The data showed that the frequencies and absolute numbers of Tfh cells were significantly lower in CD38-deficient mice as compared with B6 WT cGVHD mice throughout the experiment (**Figures 1C, D** **)**. Decreased frequencies and numbers of CD4^+^TCR-β^+^ T cells in *Cd38^−/−^* cGVHD mice were only shown 4 weeks after the adoptive transfer experiment (**Figures 1E, F** **)**. However, both WT and *Cd38^−/−^* recipients developed similar levels of splenomegaly as judged by a similar increase in the total number of splenocytes (**Figure 1G**). In contrast, the increase in spleen size was more modest in *Cd38^−/−^* than in WT mice, particularly at 8 weeks of the adoptive transfer of bm12 cells, where these differences reached statistical significance (**Figure 1H**). Since in this cGVHD model it is well stablished that the initial expansion of Tfh cells comes from the donor T cells, and to a very low extent from host T cells (18, 36), our results suggested that the distinct allo-response elicited by donor bm12 T cells in *Cd38^−/−^* recipient mice was dependent on the host cellular environment.

### Decreased Frequencies of GC B Cells and Plasma Cells Upon Induction of cGVHD in *Cd38^−/−^* Recipients

**Figure 2:**
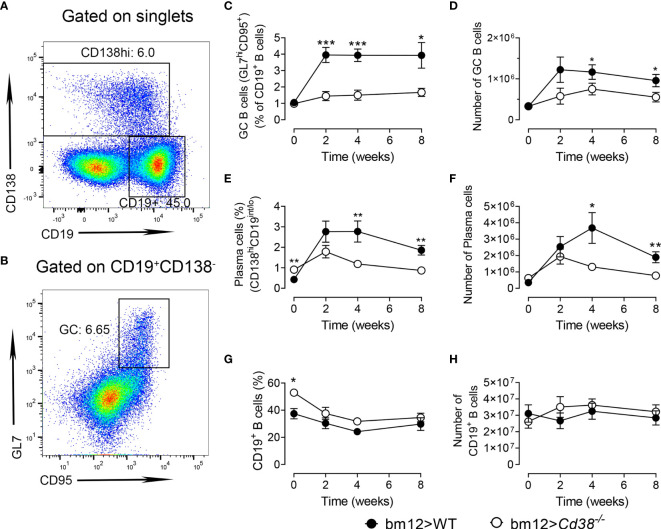
Decreased frequencies of GC B cells and plasma cells upon induction of chronic graft-*versus*-host disease (cGVHD) in *Cd38^−/−^* recipients. WT (closed circles) and *Cd38^−/−^* (open circles). **(A)** Representative flow cytometry plot showing CD19^lo-int^CD138^hi^ plasma cells and CD19^+^CD138^−^ B cells from the spleen cells gated on singlets. **(B)** Flow cytometry plot of CD95^+^GL7^hi^ GC B cells from gated CD19^+^CD138^−^ cells. **(C)** Percentages of GC B cells (of CD19^+^ B cells). **(D)** Total number GC B cells. **(E)** Percentages of plasma cells in spleen cells. **(F)** Total number of plasma cells in spleen cells. **(G)** Percentages of CD19^+^ B cells in the spleen. **(H)** Total number of CD19^+^ B cells over time upon the adoptive transfer of bm12 cells. In panels **(C–H)** WT mice (closed circles) and *Cd38^−/−^* mice (open circles). The symbols are the mean values and the vertical bars represent ± SEM. *P*-values are shown for Welch’s *t*-test. The results are cumulative data from two to four different experiments per time point and mouse type, each with three to five mice per experiment. *P < 0.05, **P < 0.01, ***P < 0.001.

Tfh and GC B cells are reciprocally supportive of each other: the frequency of Tfh is associated with the magnitude of the B-cell response, and GC B cells have been shown to promote Tfh expansion (36–38). In **Figures 2A, B**, the gating strategies used to detect plasma cells and GC B cells are shown, respectively. The whole gating strategy is shown in **Figure S2**. In agreement with the data on Tfh cells, the expansion of GC B cells (CD19^+^Fas^+^GL7^+^) in *Cd38^−/−^* recipient mice was significantly lower than that observed in WT recipient mice (**Figures 2C, D** **)**. Moreover, the percentages and numbers of plasma cells, as assessed by the high expression of CD138 in CD19^int/low^ cells, were significantly reduced in *Cd38^−/−^ versus* WT cGVHD mice 4 and 8 weeks after the adoptive transfer of bm12 spleen cells and not after 2 weeks (**Figures 2E, F** **)**. Therefore, the observed reduction in B-cell responses in the *Cd38^−/−^* cGVHD mice could result from changes in reciprocal Tfh and B-cell interactions, rather than a sole effect of the CD38 deficiency on B cells. However, both WT and *Cd38^−/−^* mice were adoptively transferred with bm12 CD38-suficient spleen cells that contain the same initial numbers of allo-reactive donor bm12 CD4^+^ T cells, which were fully functional. Moreover, WT and *Cd38^−/−^* recipient mice share the same class II I-A^b^ allele, and the frequencies and absolute numbers of CD19^+^ B cells did not change significantly from 2 to 8 weeks after the adoptive transfer (**Figures 2G, H**). In summary, the lower frequency of GC B cells in *Cd38^−/−^* recipients compared with WT might be a direct consequence of a defective allogeneic cell response of the *Cd38^−/−^* B-cell counterpart, not from defective signals provided by the donor bm12 CD4^+^ T cells.

### Decreased Frequencies and Numbers of CD11c^hi^T-bet^+^ B Cells in *Cd38^−/−^* cGVHD Mice

**Figure 3:**
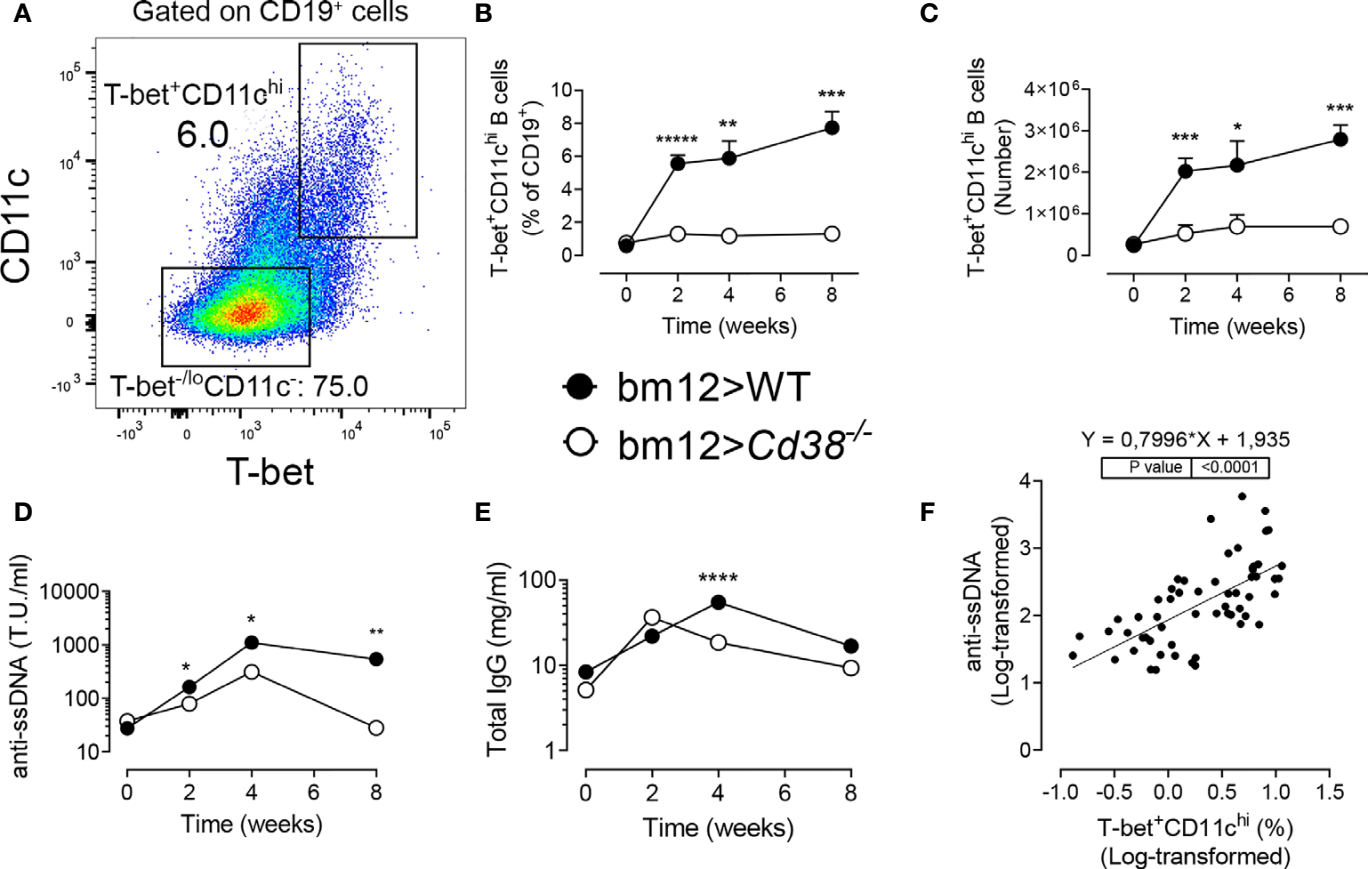
**(A–C)** Decreased frequencies and numbers of T-bet^+^CD11c^hi^ B cells in *Cd38^−/−^* cGVHD mice. **(D–F)** Decreased anti-ssDNA autoantibody serum levels in *Cd38^−/−^* cGVHD mice. Correlation with the frequencies of T-bet^+^CD11c^hi^ B cells. **(A)** Representative flow cytometry plot showing T-bet^+^CD11c^hi^ cells and T-bet^−/lo^CD11c^−^ on gated CD19^+^ B cells from spleen. **(B)** Percentages of CD11c^hi^T-bet^+^ cells (of CD19^+^ B cells). Mean ± SEM. *P*-values are shown for Welch’s *t*-test. **(C)** Total number of CD11c^hi^T-bet^+^ cells. Mean ± SEM. *P*-values are shown for Welch’s *t*-test. **(D)** Anti-ssDNA serum levels. Median values. *P*-values are shown for Mann–Whitney test. **(E)** Total IgG serum levels. Median values. *P*-values are shown for Mann–Whitney test. **(F)** Positive correlation between anti-ssDNA serum levels from cGVHD mice and the frequencies of CD11c^hi^T-bet^+^ B cells (simple linear regression). The anti-ssDNA levels and frequencies are shown in log scale. The results are cumulative data from two to four different experiments per time point and mouse type, each with three to five mice per experiment. *P < 0.05, **P < 0.01, ***P < 0.001, ****P < 0.0001, *****P < 0.00001.

B-cell-intrinsic expression of T-bet is required for the development of autoantibody-mediated disease in lupus mouse models, including in the cGVHD model (39, 40). Therefore, it was of interest to assess whether the frequencies of CD11c^hi^T-bet^+^ B cells in *Cd38^−/−^* cGVHD mice were reduced as compared with WT cGVHD mice. **Figures 3A** and **S3** illustrate the gating strategy used to identify the CD11c^hi^T-bet^+^ cells within the CD19^+^ B-cell subset. These cells were also positive for CD183 (CXCR3) on the cell surface, whose expression depends on the transcriptional activity of T-bet (**Figure S3**). There was a significant decrease in the frequencies and numbers of CD11c^hi^T-bet^+^ B cells in *Cd38^−/−^* cGVHD (**Figures 3B, C** **)**. This decrease in CD11c^hi^T-bet^+^ B cells appeared to be long-lasting as the differences with WT cGVHD mice were observed up to 8 weeks after the induction of the disease. Moreover, the weak expansion of these cells in *Cd38^−/−^* cGVHD mice was even more dramatic than in GC B cells.

### Decreased Anti-ssDNA Autoantibody Serum Levels in *Cd38^−/−^* cGVHD Mice

In *Cd38^−/−^* cGVHD mice, the anti-ssDNA levels were significantly lower than those in WT recipient mice along the study (**Figure 3D**). Total IgG, which reflects polyclonal B-cell activation, followed different kinetics in *Cd38^−/−^* cGVHD mice, with a peak 2 weeks after the adoptive transfer, diminishing steadily afterwards (**Figure 3E**). In contrast, in WT cGVHD mice, the IgG peak was at 4 weeks. It is worthy to note the significant correlation between anti-ssDNA serum levels and the frequencies of CD11c^hi^T-bet^+^ B cells (**Figure 3F**).

### Normal Expansion of Tfr Cells and Treg Cells in *Cd38^−/−^* cGVHD Mice

**Figure 4:**
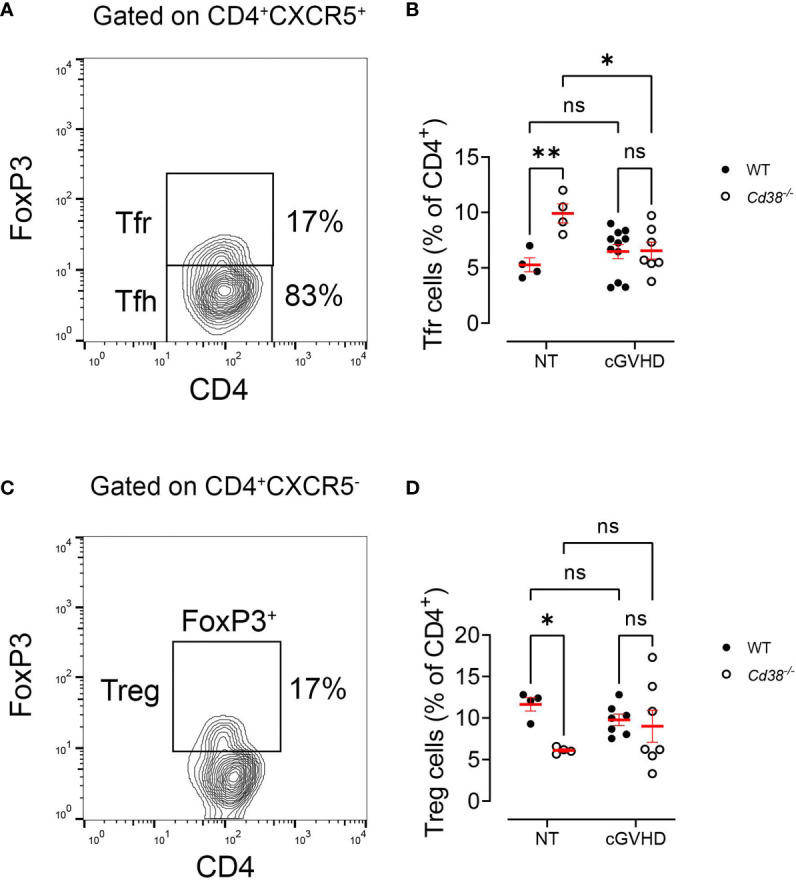
Normal expansion of Tfr cells and Treg cells in *Cd38^−/−^* cGVHD mice. **(A, C)** Gating strategy used to detect total Tfr cells (independently of PD-1 expression) and Treg cells. **(A)** Representative flow cytometry plot showing FoxP3^+^ Tfr cells and FoxP3^−^ Tfh cells on gated CD4^+^CXCR5^+^ T cells. **(B)** Frequencies of Tfr cells (% of CD4^+^) in non-treated (NT) mice or after 2 weeks of i.p. injection of bm12 cells (cGVHD). **(C)** Representative flow cytometry plot showing FoxP3^+^ Treg cells on gated CD4^+^CXCR5^−^ T cells. **(D)** Frequencies of Treg cells (% of CD4^+^) in NT mice or after 2 weeks of i.p. injection of bm12 cells (cGVHD). In panels **(B)** and **(D)** WT mice (closed circles) and *Cd38^−/−^* mice (open circles). Horizontal bars represent mean values and vertical bars ± SEM. The *P*-values are for two-way ANOVA test for multiple comparisons (Fisher’s LSD test). ns, not significant (*P* > 0.05), *P < 0.05, **P < 0.01.

Regulatory T cells (Treg) have been demonstrated to play vital roles in suppressing cellular and humoral immune responses, i.e., by suppressing autoreactive B-cell functions and subsequent autoantibody production (41, 42). Tfr cells are a specialized subset of effector Treg cells that gain access to the GC and suppresses B-cell responses. Like Tfh cells, Tfr cells express high levels of CXCR5 (which directs them to the GC), PD-1, and ICOS (43–45). However, Tfr cells originate from natural Treg precursors unlike Tfh cells, which originate from naive CD4^+^ T cells. Tfr cells also differ from Tfh cells by expressing FoxP3 and Blimp-1. Importantly, Tfr cells specifically and potently suppress both Tfh and B cells in the GC reaction (45). The low frequencies and numbers of Tfh cells (**Figures 1C, D** **)** and GC B cells (**Figures 2C, D** **)** found in cGVHD *Cd38^−/−^* mice suggested that Tfr cells may have increased presence in the GC of these mice. On the other hand, it is important to note that in CD38-deficient mice, Treg and iNKT cells are highly sensitive to NAD-induced cell death activated by ADP ribosyltransferase-2 (ART2)-mediated ADP ribosylation of P2X7 receptors (46, 47). The relatively low numbers of these immunoregulatory CD4^+^ T-cell populations greatly affect the outcome of a number of autoimmune or inflammatory diseases in CD38-deficient mice (48–50). Therefore, it was of interest to test whether the Treg cells may have a role in the weak allogeneic response triggered by the i.p. injection of bm12 cells into CD38-deficient mice, at the peak of the GC formation (41, 47). Tfr cells were defined as FoxP3^+^ within the CD4^+^CXCR5^+^ subset (**Figure 4A**), which also contains the Tfh cells (CD4^+^CXCR5^+^FoxP3^−^), whereas Treg cells were the FoxP3^+^ cells within the CD4^+^CXCR5^−^ subset (**Figure 4C**). The whole gating strategy is shown in **Figure S4**. According to Sage et al. (45), this strategy better reflects the total number of Tfr cells, independently of the expression of PD-1, and allows a better perception of the development of Tfr cells *versus* the expansion of precursor Treg cells (43, 45). Notably, the percentages of Tfr cells in *Cd38^−/−^* mice were significantly higher than in WT mice under steady-state conditions (**Figure 4B**), while the opposite occurs for Tregs (**Figure 4D**). Two weeks after the adoptive transfer, both mice showed similar frequencies of Tfr cells and Treg cells, despite some changes occurred relative to basal levels (**Figures 4B, D** **)**. Overall, these results indicate that in *Cd38^−/−^* cGVHD mice, the Treg-dependent Tfr expansion was quite similar to that in WT cGVHD.

### Distinct Kinetic Profiles of PD-1^+^ and PD-1^−^ Tfr Cells in cGVHD *Cd38^−/−^ Versus* cGVHD WT Mice

**Figure 5:**
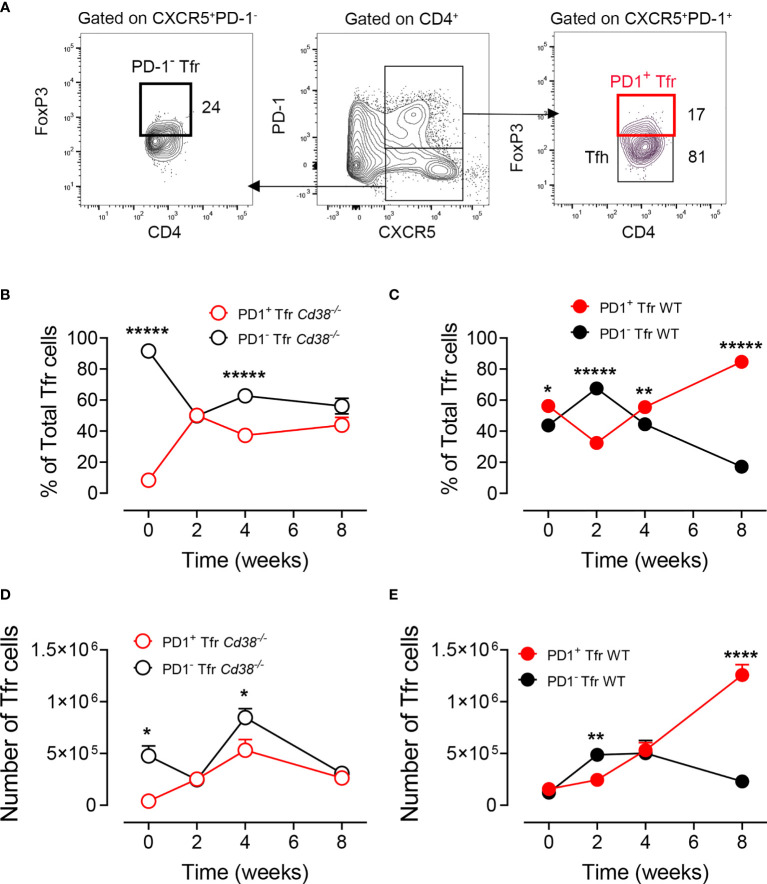
Distinct kinetic profiles of PD-1^+^ and PD-1^−^ Tfr cells in cGVHD *Cd38^−/−^ versus* cGVHD WT mice. **(A)** Gating strategy to detect PD-1^hi^CXCR5^+^ FoxP3^+^ or PD1^−^CXCR5^+^FoxP3^+^ Tfrs cells in spleen cells from cGVHD mice. Left panel: FoxP3 *versus* CD4 plot of gated CD4^+^CXCR5^+^PD-1^−^ cells, showing the frequency of the PD-1^−^FoxP3^+^ Tfr cells. Middle panel: PD-1 *versus* CXCR5 plot on gated CD4^+^ cells showing the gated CXCR5^+^PD-1^hi^ and CXCR5^−^PD-1^−^ subpopulations. Right panel: FoxP3 *versus* CD4 plot showing the frequencies of PD-1^+^FoxP3^+^ Tfr cells and PD-1^−^FoxP3^−^ Tfr cells. **(B)** Kinetics of the frequencies of PD1^+^ (open red circles) and PD-1^−^ (open black circles) Tfr cells relative to total Tfr cells in spleen cells from cGVHD *Cd38^−/−^* mice. **(C)** Kinetics of the frequencies of PD1^+^ (closed red circles) and PD-1^−^ (closed black circles) Tfr cells relative to total Tfr cells in spleen cells from cGVHD WT mice. **(D)** Kinetics of total numbers of PD1^+^ (open red circles) and PD-1^−^ (open black circles) Tfr cells relative to total Tfr cells in spleen cells from cGVHD *Cd38^−/−^* mice. **(E)** Kinetics of total numbers of PD1^+^ (closed red circles) and PD-1^−^ (closed black circles) Tfr cells relative to total Tfr cells in spleen cells from cGVHD WT mice. The symbols are the mean values and the vertical bars represent ± SEM. *P*-values are shown for Welch’s *t*-test. The results are cumulative data from two to three different experiments per time point and mouse type, each with three to four mice per experiment. *P < 0.05, **P < 0.01, ****P < 0.0001, *****P < 0.00001.

Increased PD-1 expression on Tfr cells makes them less suitable to perform their suppressive function (45); therefore, it was of interest to assess whether there were differences in PD-1 expression in Tfr cells from cGVHD *Cd38^−/−^* mice *versus* WT mice. In **Figures 5A** and **S1**, the gating strategy used to detect PD1^+^ and PD-1^−^ Tfr cells is shown. In non-treated *Cd38^−/−^* mice, 90% of the Tfr cells were PD-1^−^, with a significant decrease to about 50% 2 weeks after the adoptive transfer of bm12 cells, remaining in that range thereafter (**Figure 5B**). In contrast, in cGVHD WT mice, about 40% of the Tfr cells were PD-1^−^, with a significant increase at 2 weeks, dropping down to 17% at 8 weeks, where 84% Tfr cells were PD-1^+^ (**Figure 5C**). Absolute numbers of PD-1^+^ and PD-1^−^ Tfr cells reflected distinct kinetic profiles in *Cd38^−/−^* mice *versus* WT mice. In cGVHD *Cd38^−/−^* mice, the number of Tfr cells reached a maximum at 4 weeks with some predominance of the PD-1^−^ Tfr cells, while in cGVHD WT mice, there was a progressive increase in the number of PD-1^+^ Tfr cells over the PD-1^−^ Tfr cells that reached statistical significance only 8 weeks after the adoptive transfer of bm12 cells (**Figures 5D, E** **)**. Therefore, there was a relatively balanced situation in terms of the number of cells of each phenotype in both cGVHD *Cd38^−/−^* mice and WT mice, which is clearly broken at 8 weeks.

### Distinct Clustering of *Cd38^−/−^* cGVHD Mice

**Figure 6:**
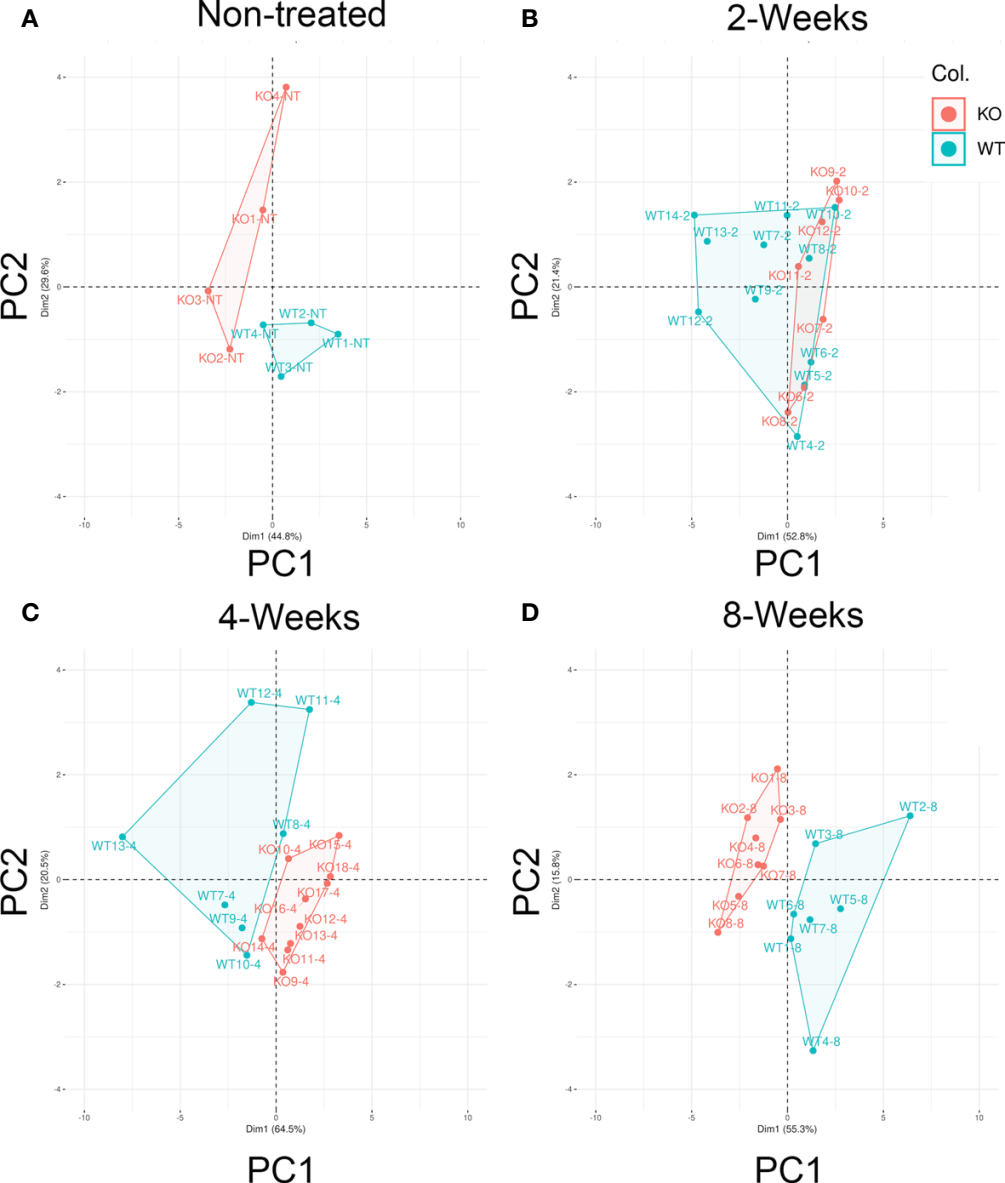
Distinct clustering of *Cd38^−/−^* cGVHD mice. The PCA score plots show the distribution of mice according with two principal components that accumulate the greatest variance (71%–85% range) of the 11 features (variables) tested. Principal component 1 (PC1, *x*-axis) X% and principal component 2 (PC2, *y*-axis) Y%. Each panel represents one given time. **(A)** Non-treated (NT) healthy mice (*n* = 4, per mouse). **(B)** Two-weeks cGVHD (*n* = 11, WT; *n* = 7, KO). **(C)** Four-weeks cGVHD (*n* = 7; WT; *n* = 10, KO). **(D)** Eight-weeks cGVHD (*n* = 7, WT; *n* = 8, KO). Each dot represents an individual mouse, and except for NT, they are representative of two or more experiments. WT samples are represented by filled red circles. *Cd38^−/−^* samples with filled green circles.

We used PCA, a multivariate statistical approach, to confirm and select the best features for distinguishing the allo-response elicited by *Cd38^−/−^* cGVHD *versus* WT cGVHD mice and to discriminate them from non-treated mice. **Figure 6** shows in four panels the score plots of the individual *Cd38^−/−^* and WT mice after the adoptive transfer of bm12 cells at different time points. Each score plot shows the distribution of mice according to the expression variance of the 11 features tested (total number of seven T-cell and B-cell subsets; anti-ssDNA, total IgG, spleen cell numbers, and spleen length). Non-treated (**Figure 6A**) and cGVHD *Cd38^−/−^* mice (**Figures 6B–D**) clustered in different areas than cGVHD WT mice with the exception at 2 weeks where 5 out of 11 WT mice overlapped with *Cd38^−/−^* mice (**Figure 6B**). This overlapping is likely due to the heterogeneity of the allo-response at that time and not to the presence of outliers (see outliers identification in the *Material and Methods*). The feature loading plot with arrows was useful to cluster each individual feature in an attempt to identify strain-specific feature expression profiles that could be used to distinguish the groups of mice analyzed (**Figure S5**). It is striking to note that most of the features seemed to be clustered to the areas where the cGVHD WT mice were located, which is in agreement with the fact that most of these features showed increased levels in these mice. In non-treated mice, the distribution of the variables was completely different, with most of them located in the upper right quadrant in an intermediate area between non-treated *Cd38^−/−^* and WT mice (**Figure S5**). Overall, these data suggest that these 11 features could be useful to discriminate the ongoing allo-immune response elicited by *Cd38^−/−^* mice *versus* WT mice. The major differences between *Cd38^−/−^* and WT mice to the bm12 challenge were observed at 4 and 8 weeks.

### Abnormal Cytokine Serum Levels in *Cd38^−/−^* cGVHD Mice

**Figure 7:**
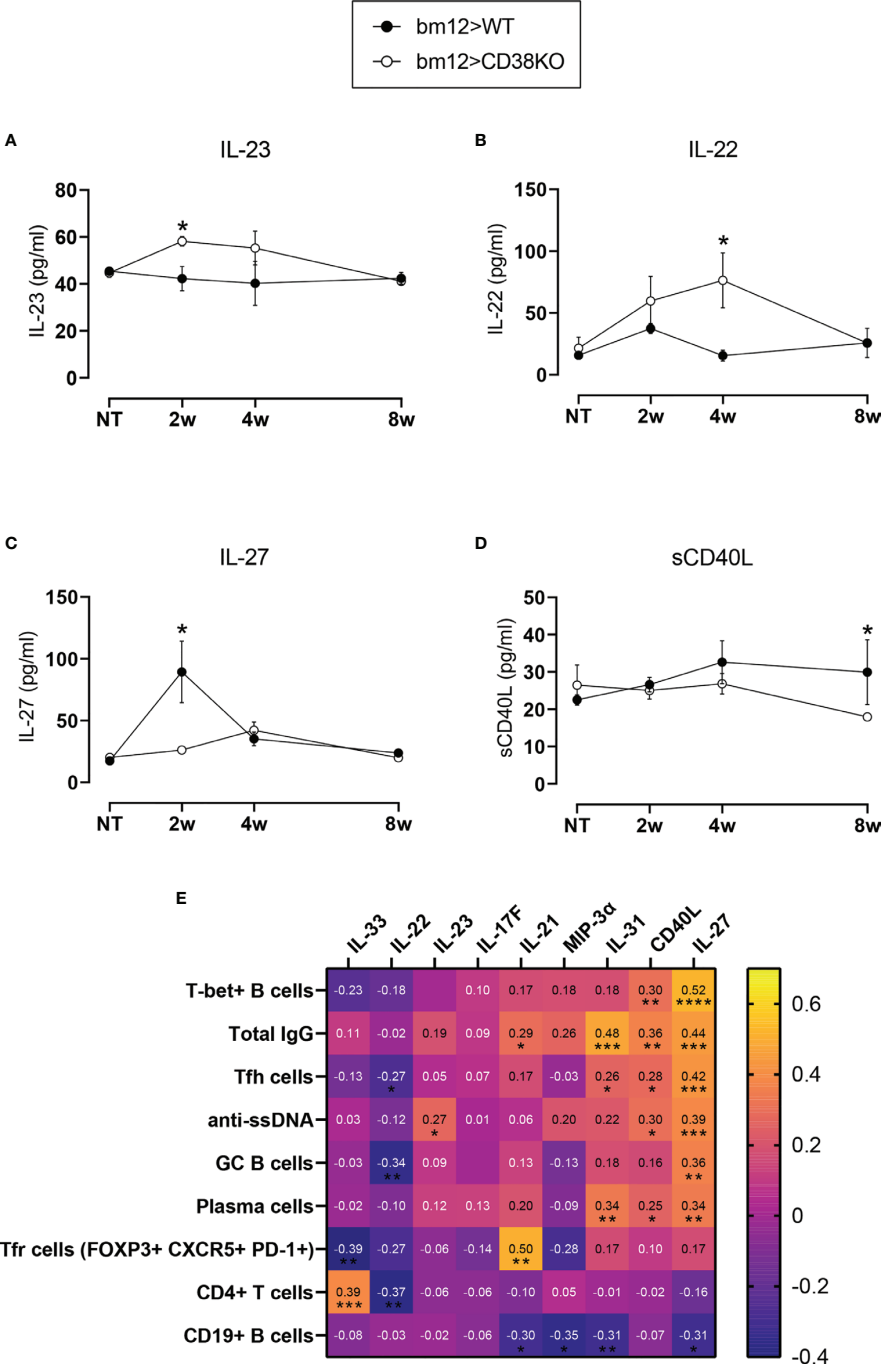
Abnormal cytokine serum levels in *Cd38^−/−^* cGVHD mice. **(A)** IL-23. **(B)** IL-22. **(C)** IL-27. **(D)** sCD40L. In panels **(A–D)** WT mice (closed circles) and *Cd38^−/−^* mice (open circles). The symbols are the mean values and the vertical bars represent ± SEM. *P*-values are shown for Welch’s *t*-test. The results are cumulative data from two to three different experiments per time point and mouse type, each with three to five mice per experiment. **(E)** Matrix showing Spearman’s *r* correlation values between cytokines serum levels (horizontal) and the frequencies of the T- and B-cell subsets analyzed + anti-ssDNA and total IgG. Asterisks below *r* numbers denote statistical significance, which could be positive correlation (no sign) or negative correlation (−). *P < 0.05.

Analysis of serum levels for 10 Th17-mediated cytokines indicated a distinct profile in *Cd38^−/−^* cGVHD mice, with significantly higher serum levels of IL-22 and IL-23 relative to WT cGVHD mice at 4 and 2 weeks, respectively (**Figures 7A, B** **)**. In contrast, IL-27 serum levels were significantly increased in WT *versus Cd38^−/−^* cGVHD mice 2 weeks after the adoptive transfer of bm12 cells (**Figure 7C**). Likewise, sCD40L serum levels were higher in WT cGVHD at 8 weeks (**Figure 7D**). Serum levels for IL-17F, IL-21, IL-31, IL-33, and MIP-3α were similar in both mouse types (**Figure S6** of the **Supplementary Material**). In a relatively small number of mice, we were also able to test another panel of cytokines including IL-10, IL-1β, IL-6, IL-17A, IFN-γ, and TNF-α. As shown in **Figure S7**, only IL-10 serum levels were significantly increased in bm12>WT mice as compared with bm12> *Cd38^−/−^* mice, 2 weeks after the adoptive transfer of bm12 cells. IL-10 has been related with autoantibody production in SLE patients (51). Notably, IL-27 correlated positively with the frequencies of most of the T-cell and B-cell subsets analyzed, with the exception of CXCR5^+^PD-1^hi^ Tfr cells and CD4^+^ T cells, while it correlated negatively with the frequencies of CD19^+^ B cells (**Figure 7E**). Moreover, IL-27 levels correlated with anti-ssDNA and total IgG levels. sCD40L followed a similar path, although with a distinct and more restrictive profile than IL-27. On the other hand, IL-22 correlated negatively with Tfh cells, GC B cells, and CD4^+^ T cells. Note that many of these correlations should be analyzed collectively and not individually. Thus, IL-27 and sCD40L correlated positively with the CD11c^hi^T-bet^+^ B-cell frequencies and IL-27, sCD40L, and IL-23 with anti-ssDNA levels (**Figure 7E**). In contrast, IL-27, IL-31, IL-21, and MIP-3α correlated negatively with the frequencies of CD19^+^ B cells (**Figure 7E**). Notable is the positive correlation found between IL-21 and the frequencies of the CXCR5^+^PD-1^hi^ Tfr cell subset and IL-33 with CD4^+^ T cells, which may be related with similar kinetic profiles.

### Proteomic Profile From Spleens of cGVHD WT Mice Reflects a STAT1-Driven Type I IFN Signature

**Figure 8:**
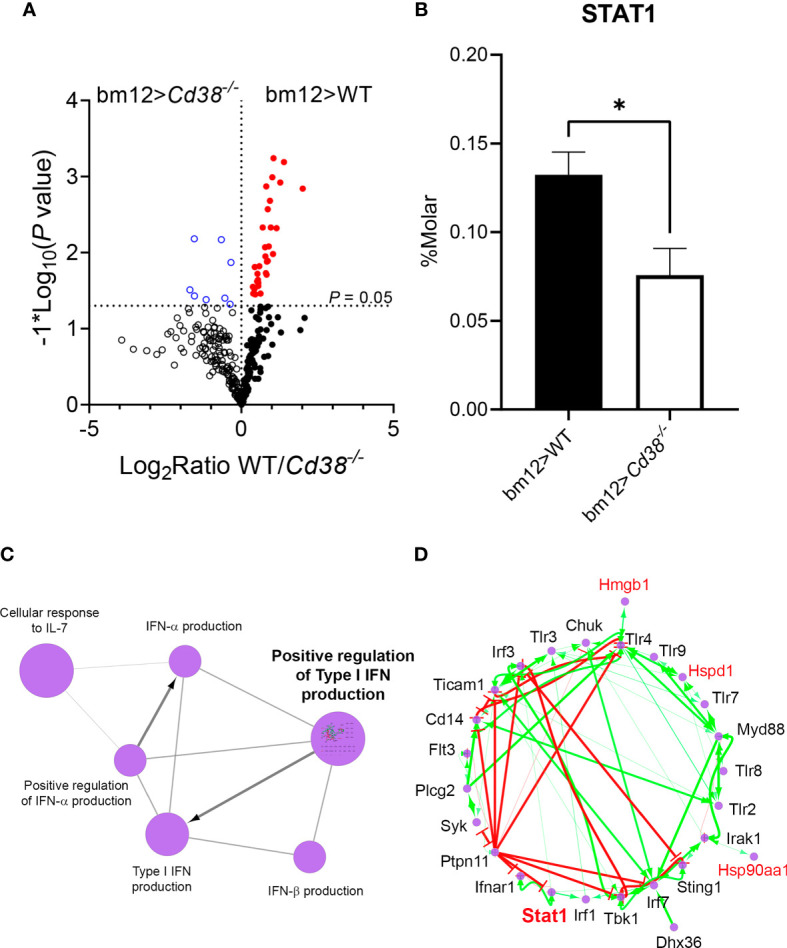
**(A)** Volcano plots showing the differences in protein abundance in spleens of bm12>*Cd38^−/−^* mice (open blue circles, upper left quadrant) relative to bm12>WT mice (closed red circles, upper right quadrant), 2 weeks after the adoptive transfer. Each circle denotes a different protein, and those above the horizontal dotted line showed statistical significant differences in abundance between mice. *P* < 0.05. **(B)** Histogram graph showing STAT1 protein abundance in spleens from bm12>*Cd38^−/−^ versus* bm12>WT mice at 2 weeks. Histograms represent the mean ± SEM, and the *P*-values are for unpaired *t*-test. Proteomic data shown in panels **(A, B)** are from two biological replicas per mouse type and three technical replicas per sample. **(C)** Functionally grouped network of GO terms/pathways and genes are visualized using the ClueGO/CluePedia application. Note that in this application the protein accession numbers of the identified proteins are transformed to their gene names. Terms (large purple circles) are linked based on *κ* score (≥0.3). Edges show the known expression. The edge thickness is scaled between the minimum and maximum scores shown. The size of the terms is related with their statistical significance. **(D)** “Positive regulation of type I interferon production’” pathway, which showed the highest statistical significance, was investigated in a subnetwork. Gene names of the identified proteins are highlighted in red. Genes not included in the initial selection are highlighted in black. Known activation [activation symbols in green (arrows)] and inhibition (inhibitory symbols in red) effects are shown. Stat1 and some of the genes of the identified proteins (highlighted in red) are acting as activators in this pathway. *P < 0.05.

The cytokine network type I IFN–IL-27–IL-10 is augmented in murine and human lupus (52), and IL-27 induces T-bet expression *via* STAT1 signaling and class switching in B cells (53). Therefore, it was of interest to test whether the augmented serum levels of IL-27 and increased T-bet expression found in B cells from WT mice had a proteomic profile sustaining these findings. To this end, a semiquantitative proteomic approach was taken using the exponentially modified protein abundance index (emPAI) and molar percentages (%M) described in the *Material and Methods* section. Protein extracts from the spleens of *Cd38^−/−^* and WT cGVHD mice and non-treated mice were analyzed. Volcano plots showed significant differences in protein abundance in the spleen lysates from bm12>*Cd38^−/−^* mice *versus* bm12>WT mice, 2 weeks after the adoptive cell transfer (**Figure 8A**). Among the proteins which showed increased abundance in spleens of bm12>WT mice *versus* bm12> *Cd38^−/−^* was STAT1 (**Figure 8B**). ClueGO functional enrichment analysis showed that STAT1 was associated with a cluster of proteins in GO terms including positive regulation of type I IFN production, type I interferon production, positive regulation of interferon-alpha production, interferon-beta production, and cellular response to IL-7 (**Figure 8C**). A positive regulation of type I interferon production was investigated in a subnetwork using the CluePedia plug-in. STAT1 was clearly involved in the regulation of the type I IFN signaling pathway with other identified proteins (**Figure 8D**). STAT1 was also inserted in other ClueGO functional terms including the IL-3 signaling pathway and the TNF-α NF-κB signaling pathway, among others (data not shown).

### Tissue Alterations in cGVHD Mice

**Figure 9:**
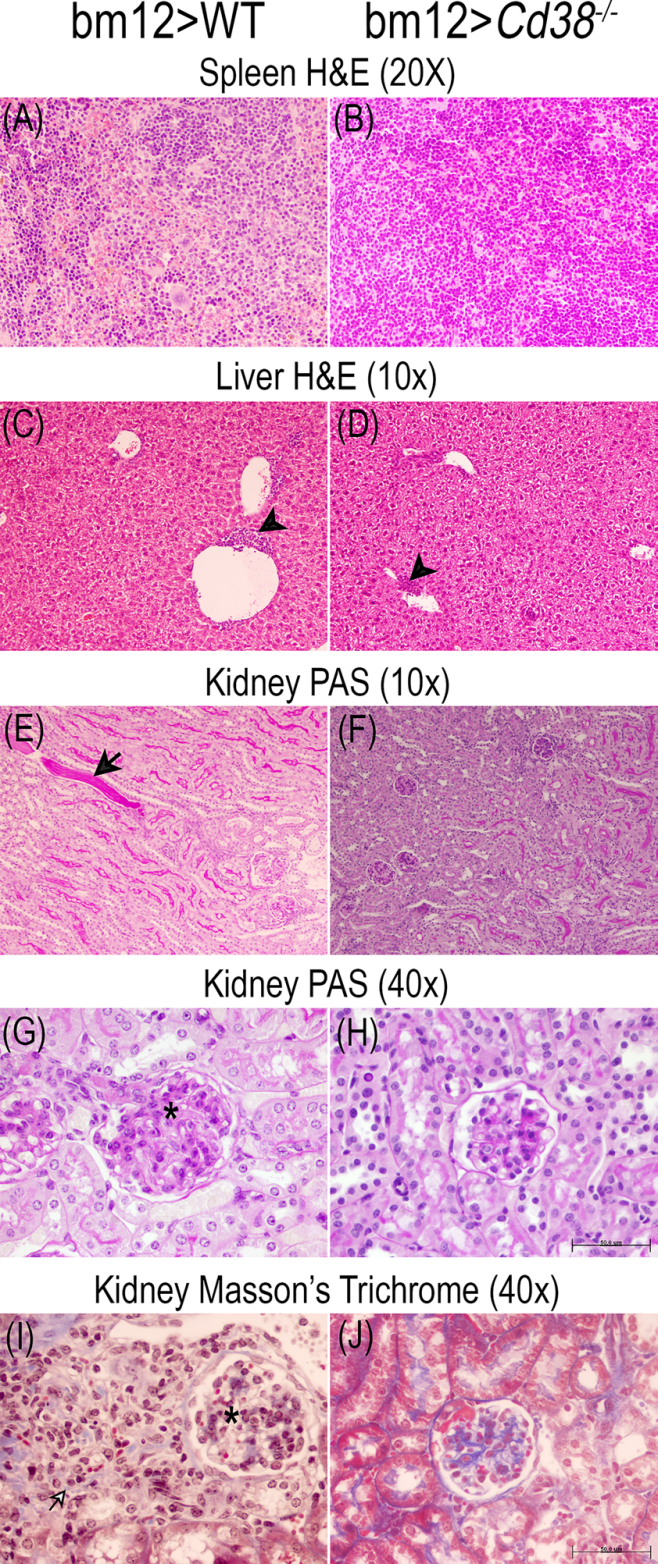
Representative microphotographs of the histological studies performed on the spleen, liver, and kidney 3-μm sections at 2 weeks of the adoptive transfer of spleen cells from female bm12 to female WT (left panels) or to *Cd38^−/−^* (right panels) mice. **(A, B)** Spleen, **(C, D)** liver, and **(E–J)** kidney. **(A–D)** Spleen and liver tissue sections stained with H&E. **(E–H)** Kidney tissue sections stained with PAS. **(I, J)** Kidney sections stained with Masson’s trichrome. Images were taken with a BH2 Olympus microscope. **(A, B)** ×20 magnification; **(C–F)** ×10 magnification; **(G–J)** 40× magnification. **(H, J)** Scale bar: 50 μm. Note in WT cGVHD mice and *Cd38^−/−^* cGVHD mice the presence of mild perivascular chronic inflammatory infiltrate in liver [black head arrow in **(C)** and **(D)**]. Note in WT cGVHD mice hyaline casts in kidney [black arrow in **(E)**], periglomerular chronic inflammatory infiltrate [black and white arrow in **(I)**], and increased glomerular size [asterisks in **(G)** and **(I)**].

**Figure 10:**
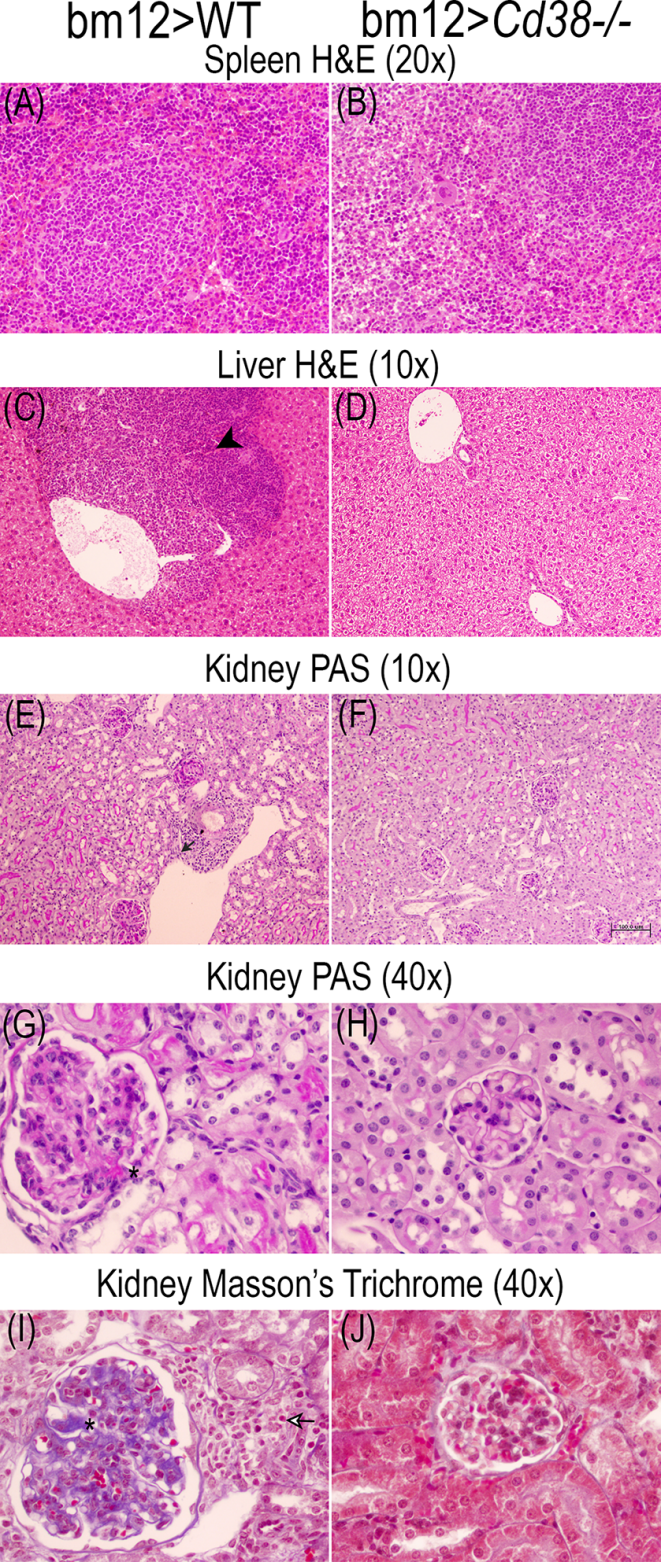
Representative microphotographs of the histological studies performed on the spleen, liver, and kidney 3-μm sections at 4 weeks of the adoptive transfer of spleen cells from female bm12 to female WT (left panels) or *Cd38^−/−^* (right panels) mice. **(A, B)** Spleen, **(C, D)** liver, and **(E–J)** kidney. **(A–D)** Spleen and liver tissue sections were stained with H&E. (**E–H**) Kidney tissue sections were stained with PAS. **(I, J)** Kidney sections were stained with Masson’s trichrome. Images were taken with a BH2 Olympus microscope. **(A, B)** ×20 magnification; **(C–F)** ×10 magnification; **(G–J)** ×40 magnification. **(F)** Scale bar: 50 μm. Note in the liver from WT cGVHD mice the presence of moderate/severe perivascular chronic inflammatory infiltrate [black head arrow in **(C)**]. Note in the kidney of WT cGVHD mice the presence of inflammatory infiltrate [small black arrow in **(E)**], periglomerular chronic inflammatory infiltrate [black and white arrow in **(I)**], and increased glomerular size with increased mesangial matrix [asterisks in **(G)** and **(I)**].

**Figure 11:**
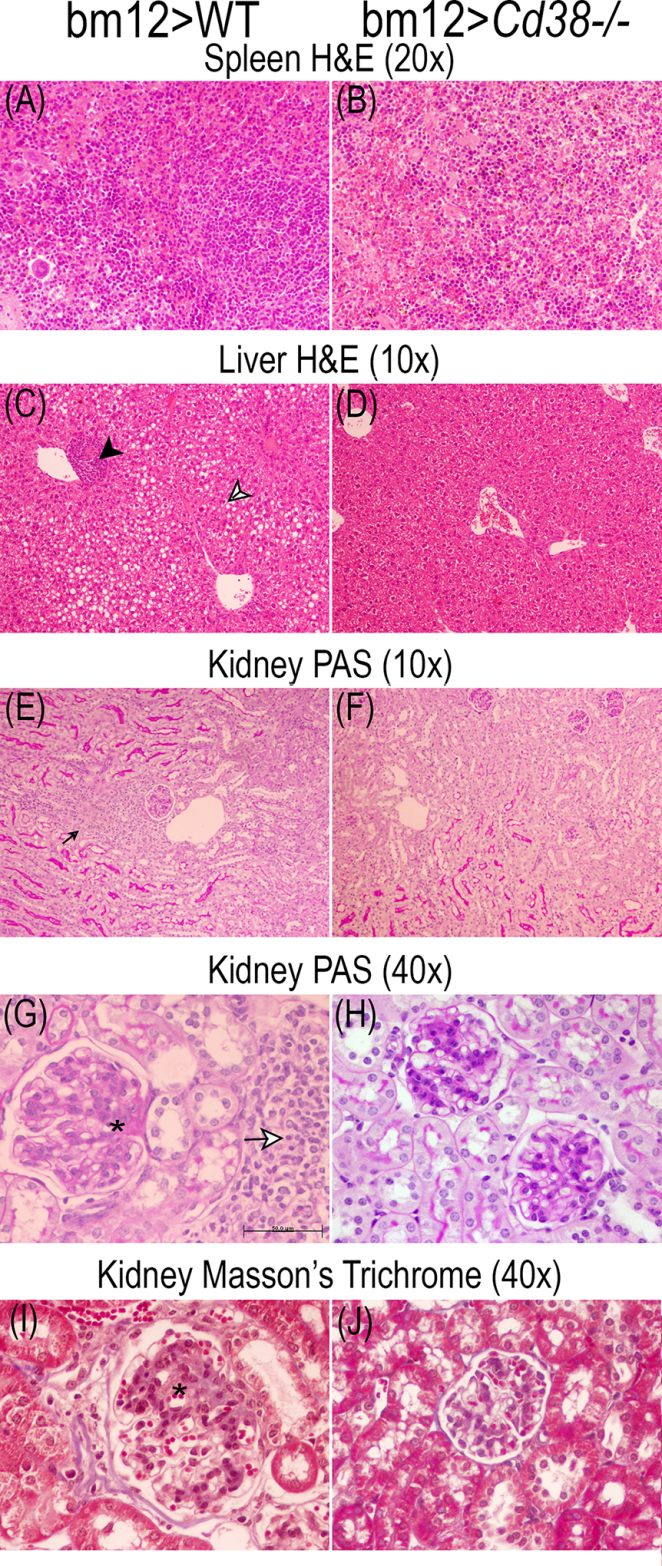
Representative microphotographs of the histological studies performed on the spleen, liver, and kidney 3-μm sections at 8 weeks of the adoptive transfer of spleen cells from female bm12 to female WT (left panels) or *Cd38^−/−^* (right panels) mice. **(A, B)** Spleen, **(C, D)** liver, and **(E–J)** kidney. **(A–D)** Spleen and liver tissue sections were stained with H&E. **(E–H)** Kidney tissue sections were stained with PAS. **(I, J)** Kidney sections were stained with Masson’s trichrome. **(A, B)** ×20 magnification; **(C–F)** ×10 magnification; **(G–J)** ×40 magnification. **(G)** Scale bar: 50 μm. Note in the liver from WT cGVHD mice the presence of mild perivascular chronic inflammatory infiltrate [black head arrow in **(C)**] and macrovesicular steatosis [black and white head arrow in **(C)**]. Note in the kidney of WT cGVHD the periglomerular chronic inflammatory infiltrate [small black arrow in **(E)**] and the increased glomerular size [asterisks in **(G)** and **(I)**].

The comparative study of the spleen, liver, and renal tissues of bm12>WT and bm12>*Cd38^−/−^* mice at 2, 4, and 8 weeks after the adoptive transfer of bm12 cells is shown in the representative microphotographs of **Figures 9** **–** **11**. In the spleen, a stronger cellular response was observed in tissue sections from bm12>WT mice as compared with bm12>*Cd38^−/−^* mice (panels **A** and **B** in each figure), with a larger number of cells, that is reflected by a significant increase in white pulp in bm12>WT *versus* bm12>*Cd38^−/−^* (for a semiquantitative assessment, see **Table S2** in the **Supplementary Material**). In the liver, greater areas of inflammatory infiltrates were detected in bm12>WT mice relative to bm12>*Cd38^−/−^* mice, with the highest intensity at 4 and 8 weeks (panels **C** and **D** of each figure). In this sense, in bm12>WT, the increased inflammation was quite patent around the centrolobular vein in the liver (indicated by a black head arrow in **Figures 9C**, **10C**, **11C**). Another striking difference in the liver of these mice at 8 weeks was the evident signs of macrovesicular steatosis in some of the bm12>WT mice (**Figure 11C**), which were not observed in liver sections from bm12>*Cd38^−/−^* mice (**Figure 11D**). No glomerular, tubulo-interstitial, or vascular lesions were present in the renal parenchyma of bm12>WT and bm12> *Cd38^−/−^* mice (panels **E**–**J** in **Figures 9** **–** **11**). However, in bm12>WT mice, PAS staining showed inflammatory infiltrates that appeared earlier and lasted longer than in bm12> *Cd38^−/−^* mice (panels **E–H**, **Figures 9** **–** **11**). The foci of inflammatory infiltrates were perivascular (**Figures 10E**, **11E**), periglomerular (**Figure 11E**), and tubulo-interstitial (**Figure 11J**), with few hyaline casts (**Figure 9E**) and without the staining evidence of the presence of glomerular immune complexes (see **Table S3** in the **Supplementary Material** for a semiquantitative assessment). Renal lesions in non-treated control groups were absent (not shown). MT staining in bm12>WT mice showed an increased thickening process of the mesangium that was maximal at 4 weeks (**Figure 10I**) and still detectable at 8 weeks (**Figure 11I**). In contrast, MT staining in bm12>*Cd38^−/−^* mice showed mesangial thickening in the glomeruli at 2 weeks (**Figure 9J**), which was markedly reduced at 4 weeks (**Figure 10J**) and virtually absent at 8 weeks (**Figure 11J**). In summary, in *Cd38^−/−^* cGVHD mice, a milder inflammatory reaction was observed in all tissues analyzed as compared with WT cGVHD, and the histological evolution of these tissue alterations suggests a faster resolution of the inflammation in *Cd38^−/−^* mice.

### Normal cGVHD Response in bm12 Mice After the Adoptive Transfer of *Cd38^−/−^* Spleen Cells

**Figure 12:**
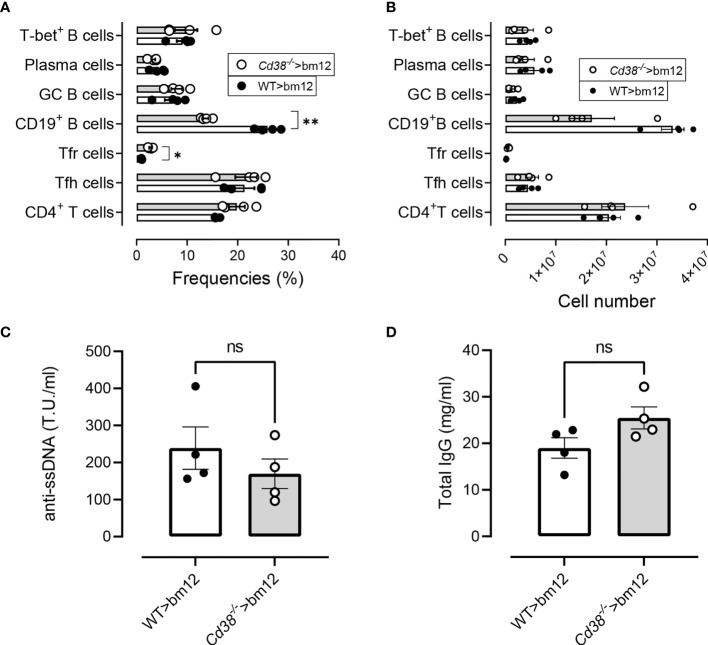
Normal cGVHD response in bm12 mice after the adoptive transfer of *Cd38^−/−^* spleen cells. **(A)** Frequencies of the T- and B-cell subsets analyzed in WT>bm12 mice (closed circles and open bars) and *Cd38^−/−^*>bm12 mice (open circles and gray bars), 2 weeks after the adoptive transfer of cells. **(B)** Total numbers of the same subsets and mice of panel **(A)**. **(C)** Anti-ssDNA serum levels. **(D)** Total IgG serum levels. Data in **(A–D)** are from one out of two independent experiments, each symbol representing one mouse. In **(A, B)**, *P*-values are for multiple *t*-tests corrected for multiple comparisons by the Holm–Šidák method. In **(C, D)**, the *P*-values are for unpaired *t*-test. ns, not significant (*P* > 0.05), *P < 0.05, **P < 0.01.

It has been described that TCR-activated *Cd38^−/−^* CD4^+^ T cells show a hybrid Th1/Th17 phenotype exhibiting intrinsically higher NAD^+^, enhanced oxidative phosphorylation, higher glutaminolysis, and altered mitochondrial dynamics that vastly improved tumor control (54, 55). These functional characteristics of *Cd38^−/−^* CD4^+^ T cells may alter the cGVHD response in bm12 mice. To test this hypothesis, we took the advantage that in the cGVHD lupus model bm12 or C57BL/6 WT mice can serve as the donor or the recipient, with similar outcomes (21). Therefore, we assessed whether the same is true using *Cd38^−/−^* cells as donors instead of being recipients. Two weeks after the adoptive transfer of *Cd38^−/−^* or WT spleen cells, bm12 recipients showed comparable increases in frequencies and absolute numbers of Tfh, GC B cells, plasma cells, and CD11c^hi^T-bet^+^ B cells (**Figures 12A, B** **)**. In contrast, increased frequencies and numbers of CXCR5^+^PD-1^hi^ Tfr cells and low frequencies and numbers of CD19^+^ B cells were shown (**Figures 12A, B** **)**, which were statistically significant for the frequencies *versus* WT>bm12 mice. Likewise, anti-ssDNA and total IgG serum levels were similar (**Figures 12C, D** **)**. Moreover, similar serum cytokine levels were observed in these mice, which is in marked contrast with the data when *Cd38^−/−^* mice were the recipients of bm12 cells (**Figure S8**).

## Discussion

In the cGVHD lupus model, the key cellular mechanism that results in the loss of B-cell tolerance is the interaction of donor CD4^+^ T cells with MHC class II on the host B-cell surface. Autoantibodies are produced almost entirely by the host B cells. The transferred donor B cells contributed neither to the autoimmune response nor to the total serum Ig, with rare exceptions (56). Therefore, in the bm12>*Cd38^−/−^* setting, the defective response of B cells is likely due to the inability of *Cd38^−/−^* B cells to respond to an allogeneic stimulus provided by donor bm12 CD4^+^ T cells. The defective B-cell response was evidenced by the low frequencies of CD11c^hi^T-bet^+^ B cells, GC B cells, and plasma cells along the disease process. These findings correlated with low levels of anti-ssDNA autoantibodies, despite the relatively strong polyclonal activation as judged by a significant increase in total IgG serum levels above basal levels. Furthermore, CD38 deficiency in host mice results in diminished generation of Tfh cells. Given that the transferred bm12-CD4^+^ T cells were CD38 sufficient and the allo-reactive donor bm12 CD4^+^ T cells activated by host MHC II provide cognate help for host B cells to initiate lupus (36), these data support the role for Ag-presenting, B-cell-intrinsic CD38 in the induction of autoreactive immune responses. Alternatively, another antigen-presenting cell such as CD8α^+^ DCs, known for its tolerogenic phenotype, may act in the early phases of the allo-immune response (57).

Another interesting feature of this model is that when the transferred donor cells were from CD38-deficient mice, the allo-immune response elicited by the interaction of donor *Cd38^−/−^* T cells with the host bm12 B cells results in similar frequencies and numbers of most T-cell and B-cell subsets analyzed in comparison with the combination WT donor>bm12 recipient. Moreover, the anti-ssDNA autoantibody response and cytokine plasma levels were quite similar to those in the WT>bm12 setting. This was an important control for the experiments where *Cd38^−/−^* mice were acting as recipients. Although in the classical cGVHD model the expansion of Tfh cells comes preferentially from the donor bm12 cells, a relatively small but significant proportion of Tfh cells come from the recipient WT mice (18). Therefore, recipient T cells may also participate somewhat in the germinal center response, as some of them do develop a Tfh phenotype. Given the fact that in the *Cd38^−/−^*donor>bm12 recipient setting, where *Cd38^−/−^* Tfh cells are strongly expanded, they seem to behave as WT Tfh cells, it is not expected that endogenous *Cd38^−/−^* Tfh cells make any functional difference in the bm12> *Cd38^−/−^* setting. To formally prove this hypothesis, it would require the use of donor and recipient mice on different congenic backgrounds, e.g., using CD45.1 bm12 donors and CD45.2 WT or CD45.2 *Cd38^−/−^* recipients in the adoptive transfer experiments would help to follow-up the fate of donor and recipient cells in the GCs and, eventually, to sort them out for functional studies.

It is worth noting that in the late phase of the allogeneic response in WT mice, the proportion and numbers of PD-1^+^ Tfr cells were significantly higher than PD-1^−^ Tfr cells, while in *Cd38^−/−^* mice, the two subsets were present in a more equilibrated situation with a ratio close to 1:1. This phenomenon occurred despite the fact that at steady-state conditions the proportion and absolute numbers of PD-1^−^ Tfr cells in *Cd38^−/−^* mice were abnormally high as compared with those in WT mice. One could argue that since the expansion of Tfh cells in *Cd38^−/−^* cGVHD mice was relatively low, while the initial expansion of Treg cells and total Tfr cells was normal, those levels of *Cd38^−/−^* Tfr cells would be suffice to efficiently inhibit Tfh cell function. It is interesting to note that the expansion of CXCR5^+^PD1^hi^ Tfr cell in WT cGVHD mice and the high expression of PD-1 make them less suitable to their suppressive function as it has been highlighted by several studies in PD-1-deficient mice (45). Moreover, the high expression of CD38 in WT B cells may suppress even further the CXCR5^+^PD1^hi^ Tfr cell function *via* adenosine-receptor signaling as it occurs in multiple myeloma tumor cells, which are highly positive for CD38 expression and where CD38 promotes tumor progression *via* the suppression of CD8^+^ T-cell function (58). In this sense, in the murine system, B-cell follicles are highly positive for CD38 expression; however, CD38 expression is low or negative in GC B cells and high in memory B cells and in other B-cell subsets (59). Therefore, during the allo-immune response in bm12>WT mice, it is feasible that the interaction of Tfr cells with CD38^+^ B cells, other than GC B cells, may occur (45).

The most striking finding of this study is the defective expansion of CD11c^hi^T-bet^+^ B cells in *Cd38^−/−^* cGVHD mice, which seems to be more severe than any other B-cell or T-cell subset analyzed, with the exception of the CXCR5^+^PD-1^hi^ Tfr cells that follow similar kinetics. Moreover, the frequency of CD11c^hi^T-bet^+^ B cells correlates with anti-ssDNA autoantibodies and serum levels of IL-27 and sCD40L. Increased serum levels of IL-27 were only detected in WT cGVHD mice. sCD40L is elevated in SLE patients, correlates with disease activity and anti-dsDNA autoantibodies, and may have the capacity to activate B cells (60, 61). Aberrant expression of CD40L in T cells might be predicted to result in activation of bystander B cells, including those that have encountered self-antigens, and to contribute to autoantibody secretion. Elevated circulating sCD40L is likely to reflect the chronic and multiclonal Th cell activation that is most characteristic of SLE (61).

IL-27 is considered an inhibitory cytokine in the differentiation of Th17 cells and induces T-bet expression *via* STAT1 signaling and class switching in B cells (53). Moreover, our label-free quantitative proteomics study demonstrates increased abundance of STAT1 in WT cGVHD mice *versus Cd38^−/−^* cGVHD mice (**Figure 8B**). *In silico* analysis of the identified proteins showed that STAT1 is functionally associated with proteins positively involved in the production of type I IFN (**Figure 8D**). In this sense, dysregulation of the IFN-I signaling pathway occurs in the bm12 cGVHD lupus-like model (19) and in the pristane lupus model (62, 63), affecting particularly B cells and autoantibody production. Thus, in the pristane model, IRF9, STAT1, and IFNAR2 are required for IgG autoantibody production and increased B-cell expression of TLR7 and TLR9 (62, 63), while in the bm12 model, type I IFN sensing by B cells decreases their threshold for BCR signaling and increased their expression of MHC class II, CD40, and Bcl-6, requirements for optimal GC B-cell functions (19). Moreover, ablation of type I IFN sensing in B cells significantly reduces the accumulation of GC B cells, plasmablasts, and autoantibodies (19), which underscores the important contribution of direct type I IFN sensing in the B-cell response and concomitant autoantibody production.

B-cell-intrinsic expression of T-bet is required for the development of autoantibody-mediated disease in lupus mouse models, including in the cGVHD model (39, 40). Likewise, several groups have reported the presence of CD11c^hi^T-bet^+^ B cells in several autoimmune diseases, including SLE. Thus, in SLE patients, the expansion of B cells lacking IgD and CD27 [double negative (DN)] reflects a subset of CXCR5^−^CD11c^+^T-bet^+^ cells (DN2), which represent the precursors of autoantibody-producing plasma cells, also termed by others as antibody-secreting cells (ASCs) (64, 65). Interestingly, in SLE patients, the differentiation of human CD11c^+^T-bet^+^ cells occurs outside the follicular zone, and therefore, it is subjected to a distinct regulation than GC B cells, which eventually also differentiate to ASCs. In contrast, most murine T-bet^+^ B cells arise from germinal centers, since most pathogens engender T-dependent immune responses, generally skewed toward Th1 (66). Consistent with this view, the T-bet^+^ B cells that accumulate with age fail to appear in CD154-deficient mice and display somatic hypermutation (67). In addition, T-bet^+^ B cells that emerge from adoptively transferred naive B cells require cell-intrinsic MHC class II and CD40 expression (67). In this sense, the cGVHD lupus model involves the adoptive transfer of bm12 T cells into mismatched MHC class II recipients and requires extensive cognate interaction between donor T cells and recipient B cells. Together, these features implicate cognate T-cell help and participation in a germinal center reaction, all hallmarks of antigen-experienced cells.

Excessive CD11c^+^T-bet^+^ B cells promote aberrant Tfh differentiation and affinity-based GC selection in murine lupus models, including the bm12 cGVHD model, through their potent antigen-presenting function (68). In this sense, ablation of B-cell-intrinsic T-bet reduces Ag presentation by B cells, diminishing T-cell activation, inhibiting spontaneous GC formation, and reducing B-cell differentiation into autoantibody-producing plasma cells (39). Triggering of the B-cell antigen receptor (BCR), IFN-γ receptor (IFN-γR), and TLR7 on B cells induces high levels of T-bet expression in humans and mice (39, 65). Given the functional association of CD38 with CD81, CD19, Lyn, Galphai-2, Hsc-70, and actin in human B cells (69) and the presence of CD38 in B-cell-derived exosomes associated with the signaling molecules CD81, Hsc-70, and Lyn (69), it would be interesting to study whether any of these receptor-mediated signaling events are affected by the absence of CD38 in B cells.

In a previous study using the pristane lupus model, we demonstrated the crucial role for CD38 in promoting aberrant inflammation and lupus-like autoimmunity *via* an apoptosis-driven mechanism, which requires TRPM2 expression (13). Mild kidney inflammation, which usually takes 2 to 3 months to develop in WT cGVHD mice, developed even milder and resolved faster in *Cd38^−/−^* cGVHD mice. This weak inflammatory reaction was also observed in other organs such as the spleen and liver. An unprecedented result was the inflammatory reaction surrounding the centrolobular vein of the liver and the evident signs of macrovesicular steatosis in some of the WT cGVHD mice, which to our knowledge has not been reported before in this lupus model.

## Conclusion

Taken together, the results of our study showed that the absence of CD38 plays a significant role in the development of the bm12 cGVHD lupus model. Although we have not identified the affected cells, all the experiments point out to a B-cell subset, with the CD11c^+^T-bet^+^ B cells being the most affected by the absence of CD38. Dysregulation of several cytokines and increased protein abundance of STAT1 were also affected. Collectively, these findings could enhance our knowledge of the pathophysiological molecular processes involved in SLE, providing strategies for early diagnosis of SLE and, in addition, therapeutic strategies based on antibodies against CD38 and pharmacological approaches targeting CD38 enzymatic activity and/or the JAK/STAT proteins.

## Data Availability Statement

The original contributions presented in the study are included in the article/**Supplementary Material**. Further inquiries can be directed to the corresponding authors.

## Ethics Statement

This study was part of the funded project SAF2017–89801-R, where all protocols and procedures involving the use of laboratory animals have been supervised and approved by the Bioethical Committee of the Consejo Superior de Investigaciones Científicas (CSIC).

## Author Contributions

JS and MZ contributed to the conception and design of the study, organized the database, and performed the experimental work. JS wrote the first draft of the manuscript. MZ wrote sections of the manuscript. RM performed the autoantibody and total IgG analyses and contributed to the interpretation of the results. NM-M and FO'V performed the histological analyses. EA-L and LT-C performed the bioinformatic analyses. MP-S-C, SR-S, and SG-F performed the flow cytometry analyses. AF-I performed the cytokine analyses. AL-S and VL-P performed the proteomics. AM-B, MD-P, MB-S, SP-C, NB-I, PC-R, AC-G, and MT-S performed the experimental work and data analysis. LM-H performed the microscopy analysis. All authors contributed to the article and approved the submitted version.

## Funding

JS and MZ received financial support through “Proyecto del Plan Estatal”: SAF2017–89801-R. The IPBLN-CSIC Proteomics Unit belonged to ProteoRed-ISCIII (PRB2; PRB3) and was supported by grants PT13/0001/0011 (IPBLN-CSIC) and PT17/0019/0010 (CIB-CSIC; IPBLN-CSIC). RM: Project: SAF2017-82905-R. FO'V: Cátedra MIS IMPLANT-UGR. The stay of AC-G in Sancho’s lab was supported by a fellowship-contract JAE-Intro (CSIC). The stay of MD-P in Sancho’s lab was supported by a 1-year post-doctoral fellowship (Reference No. 502492) from the Consejo Nacional de Ciencia y Tecnología (CONACYT) of México. EA-L was recipient of a postdoctoral fellowship from the regional Andalusian Government.

## Conflict of Interest

The authors declare that the research was conducted in the absence of any commercial or financial relationships that could be construed as a potential conflict of interest.

## Publisher’s Note

All claims expressed in this article are solely those of the authors and do not necessarily represent those of their affiliated organizations, or those of the publisher, the editors and the reviewers. Any product that may be evaluated in this article, or claim that may be made by its manufacturer, is not guaranteed or endorsed by the publisher.
