# Supplementary material for: CD38 Deficiency Ameliorates Chronic Graft-Versus-Host Disease Murine Lupus via a B-Cell-Dependent Mechanism
Source: Front Immunol. 2021 Aug 24;12:713697. doi: 10.3389/fimmu.2021.713697 (PMC8421681; doi:10.3389/fimmu.2021.713697)
Supplement: Supplementary file 1 [file DataSheet_1.pdf]

Supplementary Material

1 Supplementary Tables

Table S1. Number of experiments and number of mice per experiment

	**bm12>*Cd38*$^{-/-}$**	**bm12>WT**
**2 weeks**	Experiments= 4<br>Mice/experiment= 3±0.82<br>N=12	Experiments= 4<br>Mice/experiment= 3.25±0.96<br>N=13
**4 weeks**	Experiments= 4<br>Mice/experiment= 4.5±1<br>N=18	Experiments= 4<br>Mice/experiment= 3.25±0.5<br>N=13
**8 weeks**	Experiments= 2<br>Mice/experiment= 4<br>N=8	Experiments= 2<br>Mice/experiment=3.5±0.71<br>N=7
	***Cd38*$^{-/-}$>bm12**	**WT>bm12**
**2 weeks**	Experiments= 2<br>Mice/experiment= 4.5±0.71<br>N=9	Experiments= 2<br>Mice/experiment= 4.5±0.71<br>N=9

Number of experiments, number of mice per experiment (N±SD), and total mice (N). Non-treated *Cd38*$^{-/-}$ (N=4) and non-treated WT (N=4) mice were also analyzed.

Table S2. Histopathological analysis of spleen tissue sections stained with haematoxylin/eosin (H&E)

	bm12>WT			bm12>*Cd38*$^{-/-}$		
**Time[1] (mice)[2]**	**2w (n=7)**	**4w (n=7)**	**8w (n=7)**	**2w (n=4)**	**4w (n=15)**	**8w (n=8)**
**White pulp (%)**	55.71 ± 2.02 *	50 ± 3.78	54.29 ± 3.69	45 ± 2.89	52 ± 2.8	46.25 ± 1.83
**Red pulp (%)**	44.29 ± 2.02	50 ± 3.78	45.71 ± 3.69	55 ± 2.89 *	48 ± 2.8	53.75 ± 1.83
**Megakaryocytes[3]**	39.17 ± 8.52	41.48 ± 15.28	101.38 ± 28.75 **	80.65 ± 19.75	60.22 ± 10.14	56.45 ± 8.07
**Plasma cells[3]**	11.52 ± 9.12	6.91 ± 4.8	9.22 ± 9.22	16.13 ± 11.41	16.13 ± 6.1	12.1 ± 4.03 *
**Lymphocytes[3]**	9852.53 ± 494.3	10092.17 ± 615.22	11935.49 ± 899.1	10911.29 ± 192.369	10419.36 ± 471.61	10943.55 ± 552.03
**Apoptotic cells[3]**	43.78 ± 18.86	11.52 ± 6.78	59.91 ± 17.85 *	16.13 ± 16.13	13.98 ± 4.95	36.29 ± 9.02
**Mitosis[3]**	36.87 ± 14.81	34.56 ± 12.41	66.02 ± 17.17	32.26 ± 22.81	34.41 ± 9.29	32.26 ± 14.93

[1]Time in weeks after the i.p. injection of bm12 cells.
[2]Number of mice analyzed.
[3]A millimeter scale in the eyepiece of a microscope (BH2 Olympus (LabX, RRID: SCR_020338) with 40% objective was used to count the number of leucocytes subsets per mm^2 in spleen. Values are shown as mean ± SEM.
* The number of asterisks represent the *P* value classification and indicates the number of cero digits after the decimal of the significant *P* value. Unpaired *t* test with Welch correction.

Table S3. Semi-quantitative assessment of kidney damage

	bm12>WT			bm12>*Cd38*$^{-/-}$		
**Time[1] (mice)[2]**	**2w (n=8)**	**4w (n=7)**	**8w (n=6)**	**2w (n=4)**	**4w (n=15)**	**8w (n=7)**
**Hyaline cast[a]**	0.875 ± 0.125	1 *	0.50 ± 0.224	0.75 ± 0.25	0.60 ± 0.131	0.571 ± 0.202
**Fibrosis[a]**	0.50 ± 0.189	0.571 ± 0.202	0	1 *	0.667 ± 0.126	0
**Inflammatory infiltrate[a]**	1.125 ± 0.125 ****	1.143 ± 0.143	0.667 ± 0.211	0	1	0.375 ± 0.183
**Cells No/glomerulus[b]**	24.08 ± 1.42	25.29 ± 1.29	23.03 ± 0.88	26.85 ± 1.67	28.69 ± 1.09	25.43 ± 1.41

[1]Time in weeks after the i.p. injection of bm12 cells.
[2]Number of mice analyzed.
[a]Mean ± SEM of semi-quantitative scale (0–3).
[b]Mean ± SEM of number of cells/glomerulus per mouse.
* The number of asterisks represent the *P* value classification and indicates the number of cero digits after the decimal of the significant *P* value. Unpaired *t* test with Welch correction.
Glomerular lesions were assessed in at least 100 glomeruli (material and methods). Tubulo-interstitial damage (hyaline casts, fibrosis and inflammatory infiltrate was indicated). Injury was graded according to Shih et al., (doi: 10.1038/ki.1988.119)). A semi-quantitative scale of 0 to 3.0 was considered: 0, normal; 0.5, small focal areas of damage; 1, involvement of less than 10% of the cortex; 2, involvement of 10 to 25% of the cortex; 3, involvement above 25% of the cortex. Morphological study was done on 3-micrometer sections with light microscopy, using the most appropriate stain for each lesion (material and methods).

2 Supplementary Figures

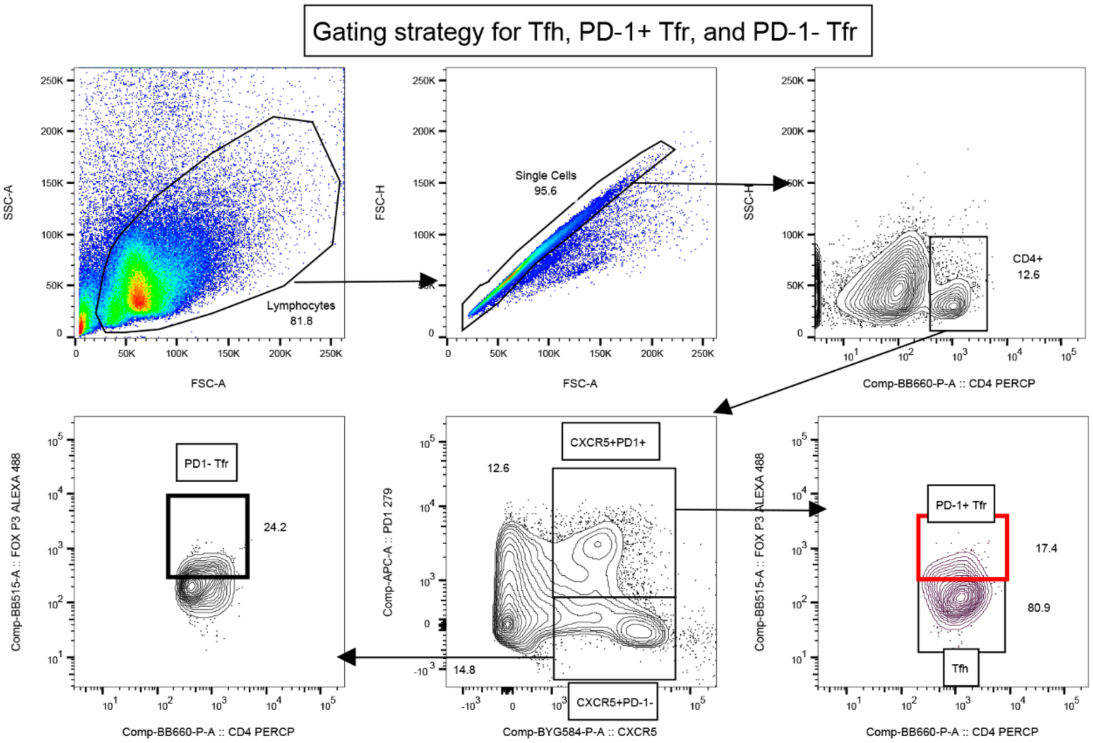

FIGURE S1 | Gating strategy for Tfh, PD-1+ Tfr, and PD-1- Tfr analysis by flow cytometry. Surface staining for the indicated markers was followed by intracellular staining of FoxP3 in permeabilized cells as described in Material and Methods. Using this strategy, the 3 cell subsets could be properly analyzed.

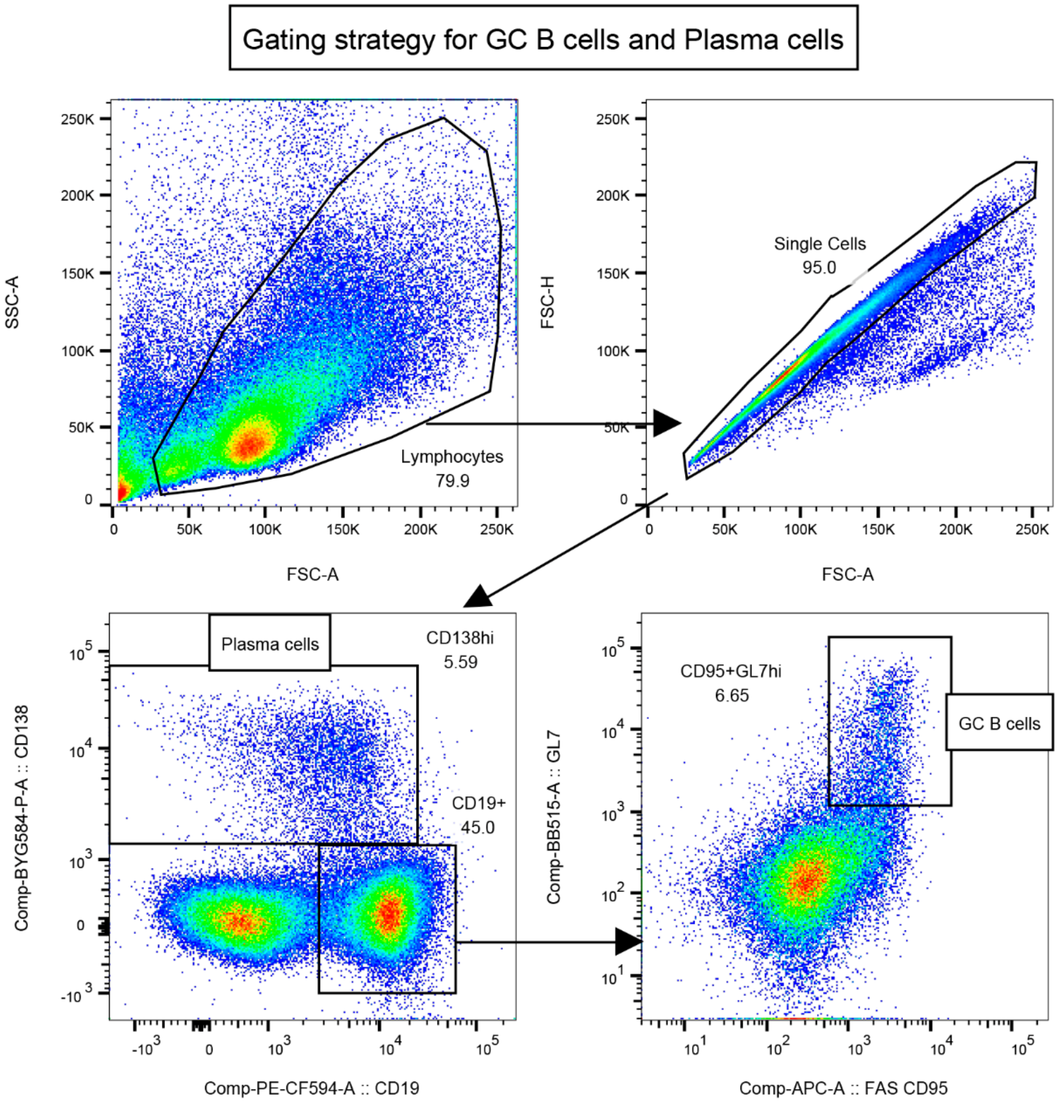

FIGURE S2 | Gating strategy for GC B cells and plasma cells analysis by flow cytometry. Surface staining for the indicated markers was performed as indicated in Material and Methods.

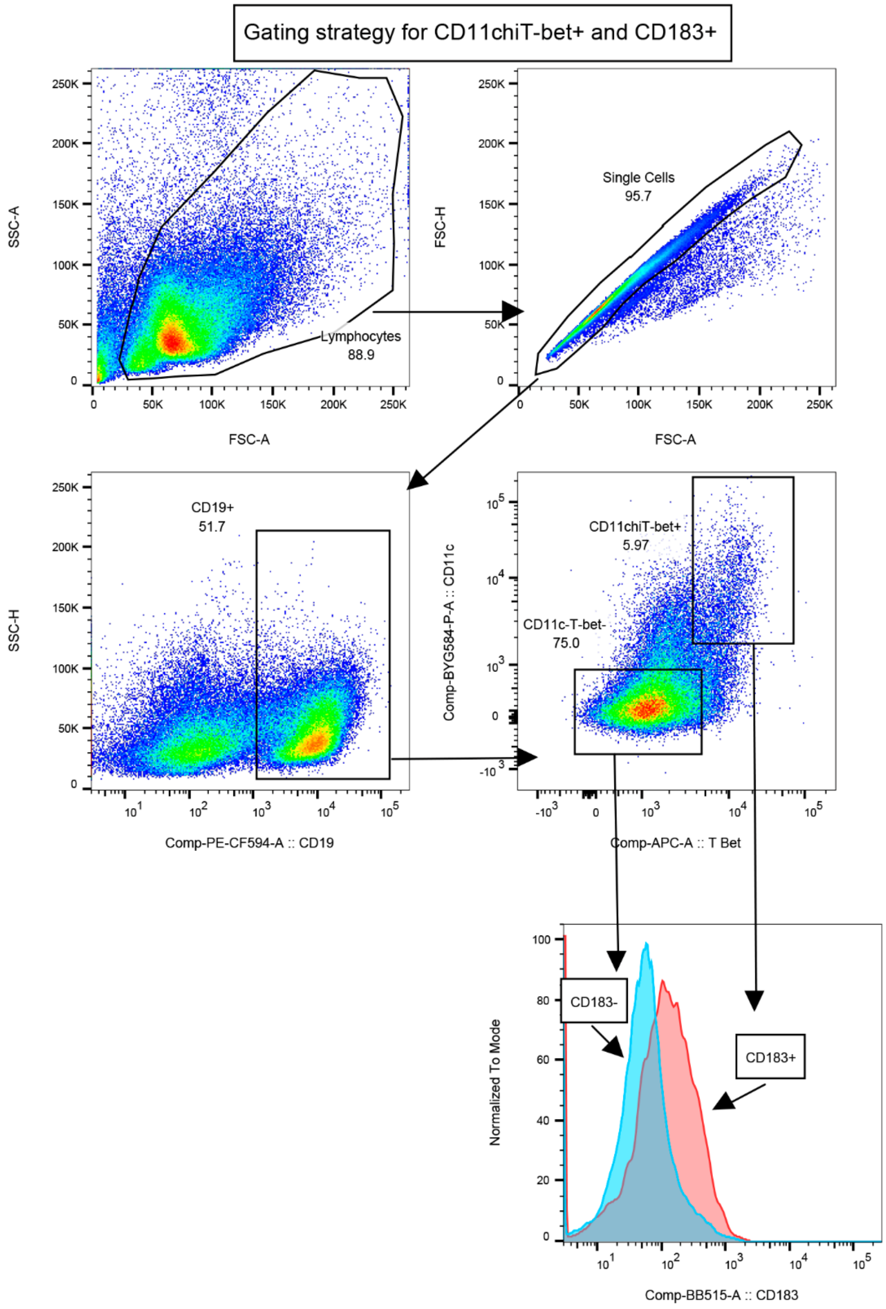

FIGURE S3 | Gating strategy for CD11c^{hi}T-bet^{+} B cells analysis by flow cytometry. Surface staining for the indicated markers was followed by intracellular staining of T-bet in permeabilized cells as described in Material and Methods.

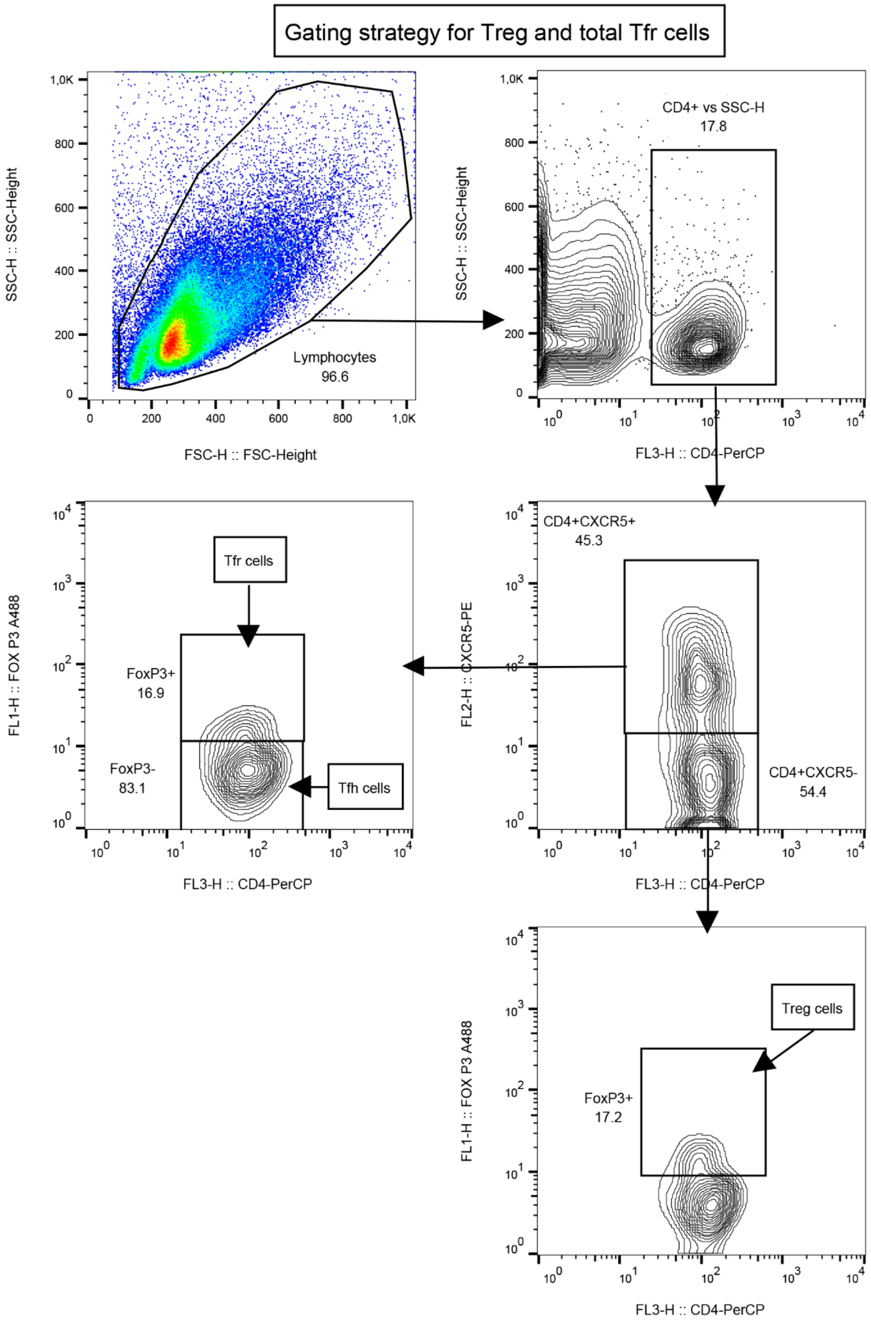

FIGURE S4 | Gating strategy for Treg and total Tfr analysis by flow cytometry. Surface staining for the indicated markers was followed by intracellular staining of FoxP3 in permeabilized cells as described in Material and Methods. Note that after gating on CD4^{+} cells, the next gating was CXCR5^{+} vs CXCR5^{-} cells to separate CXCR5^{+} Tfr from CXCR5^{-} Treg cells. With this strategy CXCR5^{+}FoxP3^{-} Tfh cells could also be analyzed.

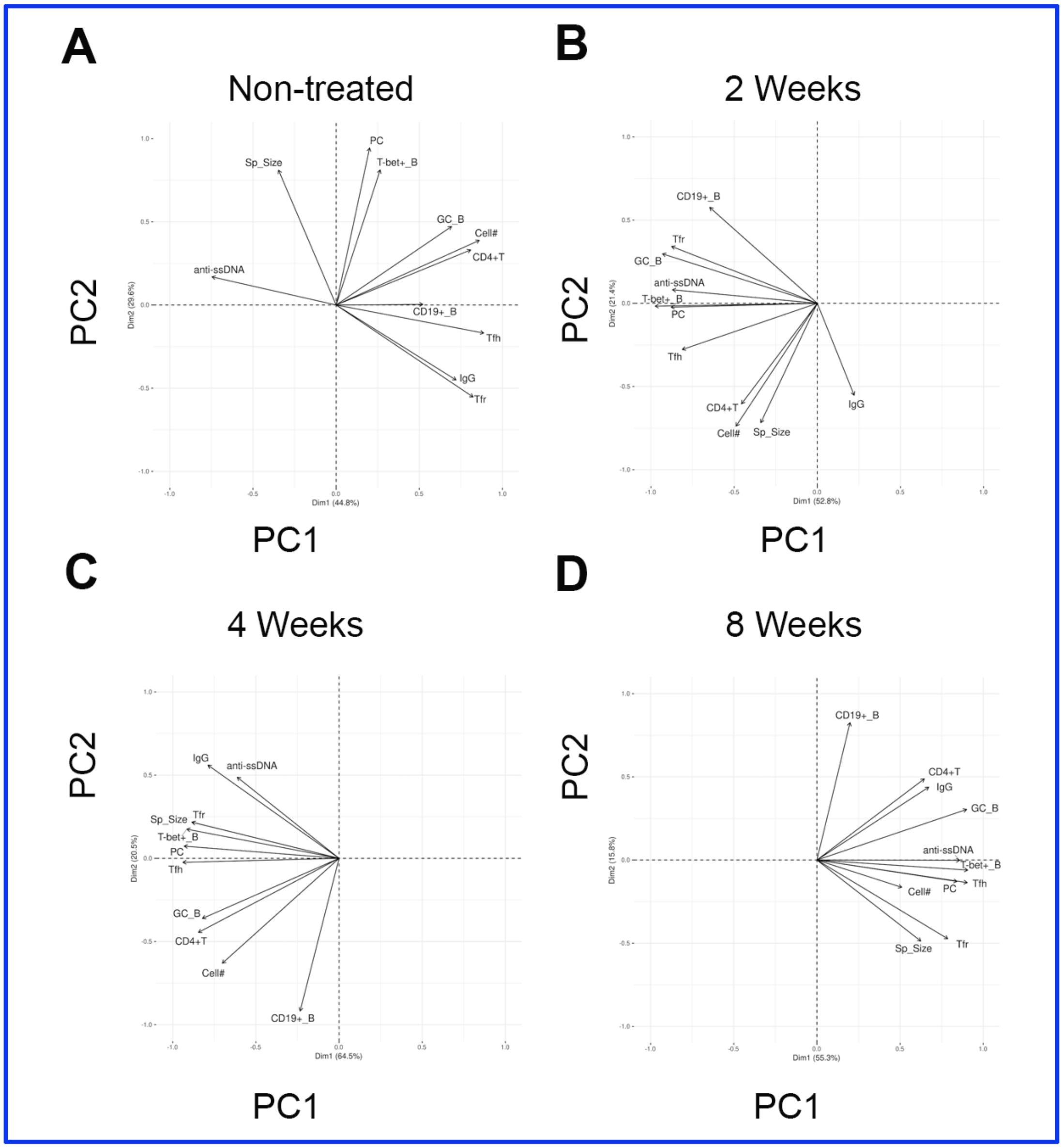

FIGURE S5 | PCA loading plots show the clustering tendencies of the 11 variables (features) used to analyze the cGVHD mice. Arrows indicate the direction of each variable within each panel corresponding to the 4 groups of mice analyzed.

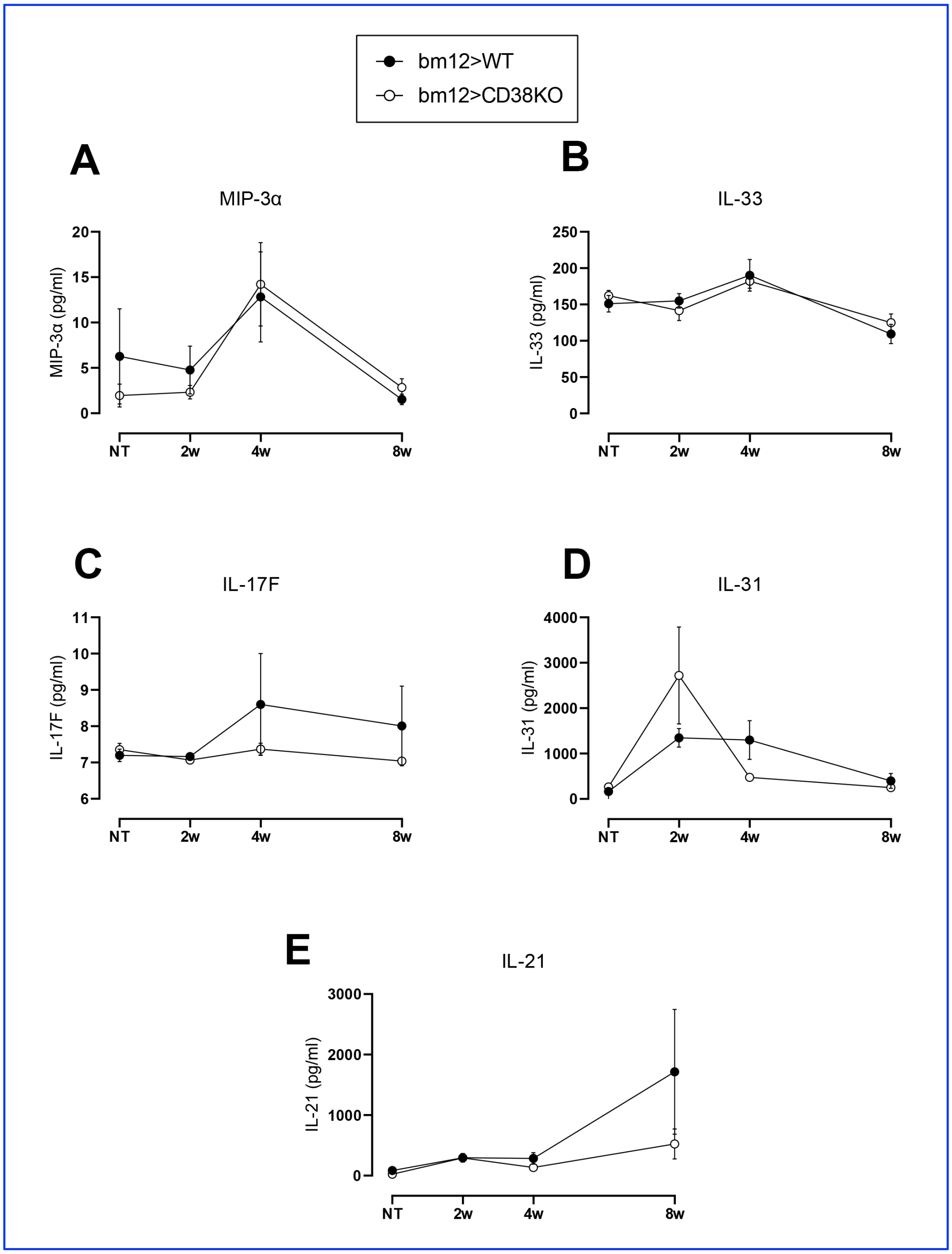

FIGURE S6 | Additional Th17 cytokine serum levels in *Cd38-/-* cGVHD mice vs WT cGVHD. **(A)** MIP-3a. **(B)** IL-33. **(C)** IL-17F. **(D)** IL-31. **(E)** IL-21 In panels **(A-E)** WT mice (closed circles), *Cd38-/-* mice (open circles). The symbols are the mean values and the vertical bars represent ± SEM. *P* values for the comparison WT vs $Cd38^{-/-}$ cytokine serum levels are not significant for Welch's *t* test. The results are cumulative data from 2-3 different experiments per time point and mouse type, each with 3-5 mice per experiment.

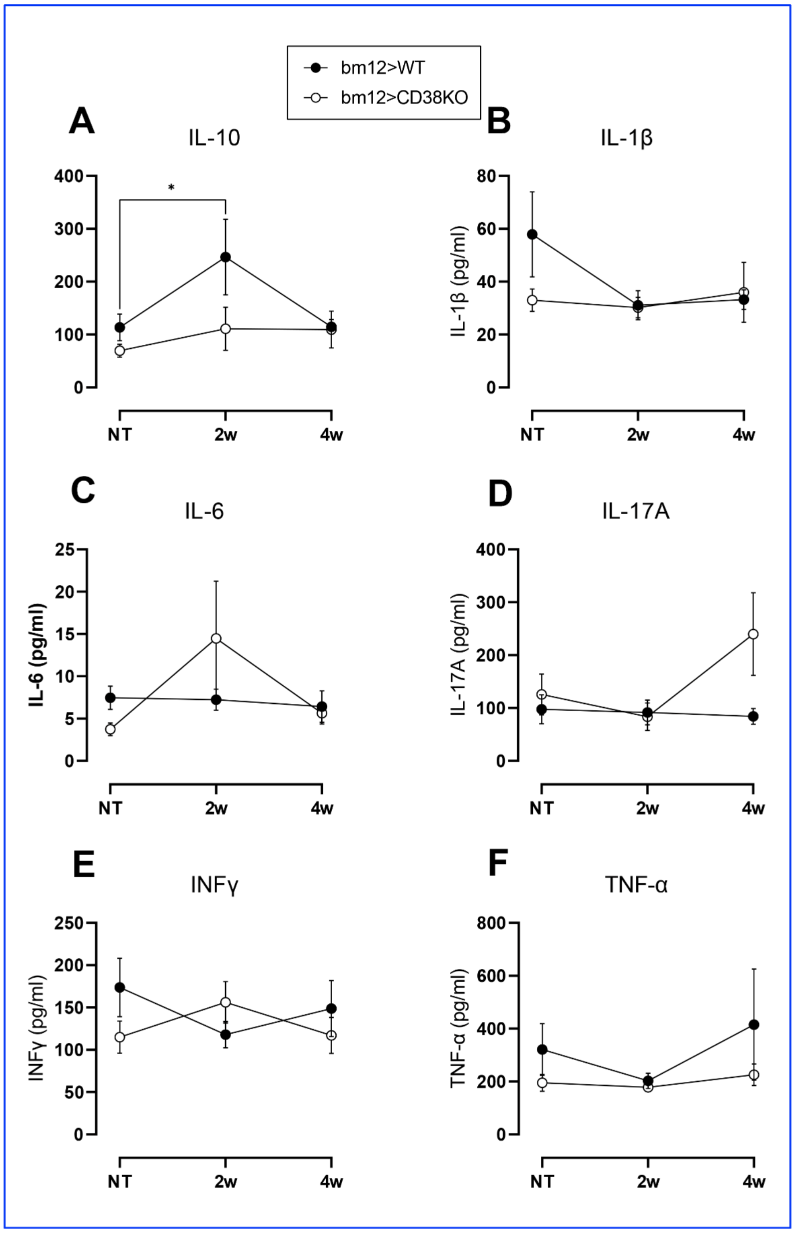

FIGURE S7 | Th1 and IL-17A cytokine serum levels in *Cd38-/-* cGVHD mice vs WT cGVHD. **(A)** IL-10. **(B)** IL-1β. **(C)** IL-6. **(D)** IL-17A. **(E)** IFN-γ. **(F)** TNF-α. In panels **(A-F)** WT mice (closed circles), *Cd38-/-* mice (open circles). The symbols are the mean values and the vertical bars represent ± SEM. *P* values are for Welch's *t* test. The results are cumulative data from 2 different experiments per time point and mouse type, each with 3-4 mice per experiment.

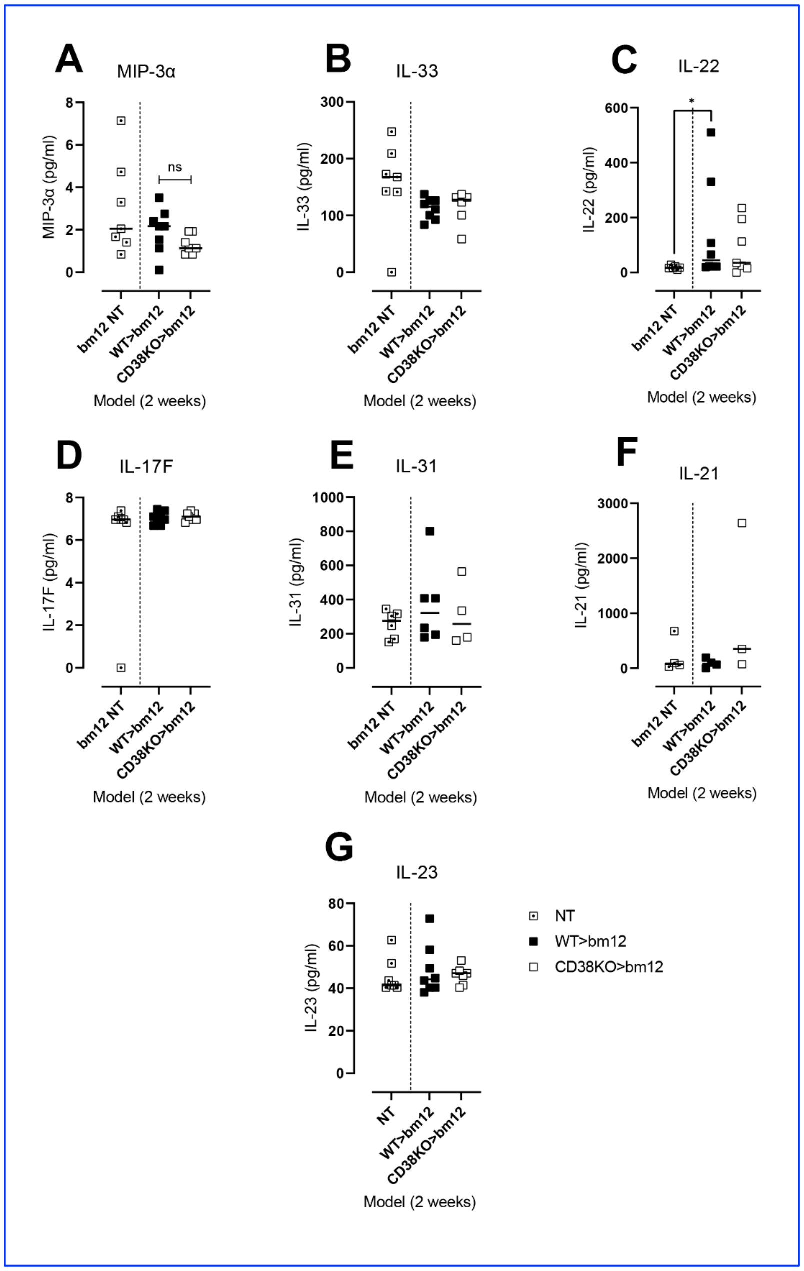

FIGURE S8 | Cytokine serum levels in *Cd38-/-*>bm12 mice vs WT>bm12 mice at 2 weeks. **(A)** MIP-3α. **(B)** IL-33. **(C)** IL-22. **(D)** IL-17F. **(E)** IL-31. **(F)** IL-21. In panels **(A-F)** WT>bm12 mice (closed squares), *Cd38-/-*>bm12 mice (open squares), and non-treated (NT) mice (open squares with a dot inside). Each symbol corresponds to a serum from an individual mouse, and the horizontal bar represents the median value. *P* values are for Mann-Whitney test. The results are cumulative data from 2 different experiments per mouse type, each with 3-4 mice per experiment.
